# The global distribution of actinomycetoma and eumycetoma

**DOI:** 10.1371/journal.pntd.0008397

**Published:** 2020-09-24

**Authors:** Darcy Emery, David W. Denning

**Affiliations:** 1 Manchester Medical School, University of Manchester, Manchester, United Kingdom; 2 The University of Manchester, National Aspergillosis Centre, Wythenshawe Hospital, Manchester Academic Health Science Centre, United Kingdom; 3 Global Action Fund for Fungal Infections, Geneva, Switzerland; Faculty of Science, Ain Shams University (ASU), EGYPT

## Abstract

**Background:**

Mycetoma, a chronic infection of the skin and underlying structures, affects those with a close relationship to the land, often in resource-poor areas of the world. Whether caused by any one of a variety of fungus or bacteria, mycetoma causes significant disability and mortality. Acknowledged as a neglected tropical disease (NTD) by the World Health Organization (WHO) in 2016, mycetoma is susceptible to being misunderstood, misdiagnosed, and mismanaged. In an effort to shift the balance in favor of recognition and effective treatment, sound epidemiological understanding is required.

**Methods and findings:**

In this paper, a literature review of case reports and series (332 papers in total) is presented as three maps. We identified 19,494 cases dating from 1876 to 2019, with cases contracted in 102 countries. The first map shows where mycetoma has ever been reported, the second shows how many cases have been reported, and the third shows the ratio of eumycetoma (fungal) to actinomycetoma (bacterial). Most cases are found in Mexico, India, and Sudan, where mycetoma is studied rigorously. We identified emergence of new geographical loci, including the United States, Venezuela, Italy, China, and Australia. Notably, mycetoma is reported far outside the tropics. In the Americas, bacterial forms dominate, whereas, in Africa and Asia, the picture is more varied.

**Conclusions:**

With better understanding of the epidemiology of mycetoma, more can be done to direct education, preventive measures, and treatment to at-risk areas, enabling a reduction in disease burden.

## Author summary

Mycetoma is an ancient and misunderstood chronic disease. Affecting the skin and subcutaneous structures, infection often occurs in the feet of land laborers and can be caused by several otherwise rare human pathogens. Traditionally seen as a tropical disease, it affects those in resource-poor regions. The best methods of diagnosis are rarely available in these areas; late diagnosis increases the need for invasive surgical treatment. Available medical treatment can be prohibitively expensive. Early diagnosis and identification of the causative agent is necessary for effective treatment. For this paper, a literature review was undertaken to find all published cases of mycetoma linked to a location and the data used to create three maps. The maps show where mycetoma has ever been reported, how many cases have been reported, and what kind of microorganism was present. We included 19,494 cases, most of which occurred in Mexico, Sudan, and India. Notably, some cases arose far outside the tropics. In the Americas, most cases are bacterial; elsewhere, the picture is more varied. Many gaps remain in the epidemiological study of mycetoma, but this paper aims to shed light on the significant reach of the disease, with a view to increasing its visibility on the global stage and, therefore, the scope for reducing the burden of this neglected tropical disease.

## Introduction

Mycetoma, a chronic [1] infection of the skin and underlying structures [2], leads to significant disability [3] and mortality [4]. It can be caused by any one of several fungi (eumycetoma) [5] or bacteria (actinomycetoma) [6], some more common than others (Table 1). As a “socioeconomically biased disease” [7], mycetoma disproportionately infects those in low-income regions, mostly affecting young male field workers [8]. Thus, significant financial strain [9] (at a societal [10] and personal level) ensues. Mycetoma was acknowledged as a neglected tropical disease (NTD) by the World Health Organization (WHO) in 2016 [11] and continues to evade efforts to estimate its true burden [12].

**Table 1:** Common and uncommon causative agents of eumycetoma and actinomycetoma [13,14].

	Fungi	Bacteria
Common agents	*Madurella mycetomatis (M*. *mycetomi) and sister species M*. *pseudomycetomatis*, *M*. *fahalii*, *M*. *tropicana* *Trematosphaeria grisea (Madurella grisea)* *Medicopsis romeroi (Pyrenochaeta romeroi)*	*Actinomadura pelletieri* *A*. *madurae* *Streptomyces somaliensis* *Nocardia brasiliensis* *N*. *asteroides*
Uncommon agents	*Neotestudina rosatii* *Scedosporium* spp. *Curvularia lunata* *Falciformispora senegalensis and F*. *tompkinsii (Leptosphaeria* spp*)* *Biatriospora mackinnonii (P*. *mackinnonii)* *Pseudochaetosphaeronema larense* *Aspergillus nidulans* *Rhytidhysteron rufulum* *Roussoella* spp. *Emarellia paragrisea*	*N*. *farcinica* *N*. *otitidiscaviarum (N*. *caviae)* *A*. *israelii*

Note that with modern taxonomic revision, especially of the agents of eumycetoma, frequency is approximate. Previous names are shown in brackets, when available.

Sometimes taking years to manifest [15], infection involves swellings [16], usually on the feet [13]. The resulting sinuses exude “grains” of fungal or bacterial matter [8]. For diagnosis, the clinical triad of a subcutaneous mass, grains, and sinuses is often used [16], supplemented by imaging and laboratory techniques [2]. In practice, many endemic regions simply do not have the financial resources to carry out these necessary tests [16].

Eumycetoma has been reported more in Africa, actinomycetoma more often in South and Central America [8]. To treat, clinicians need to distinguish between the bacterial and fungal form of mycetoma [6]; cure of both is possible [16]. For actinomycetoma, this consists of long-term combination antibiotic treatment, given in cycles [17]. Eumycetoma responds poorly to the few effective medical therapies that exist, so surgical treatment is common [18]; amputation is a last resort [19]. Antibiotics and antifungals effective in the treatment of mycetoma are included in the WHO Model List of Essential Medicines [20], but the long duration of treatment [8] and monitoring [16] means they may be prohibitively expensive [2]. In Sudan, for example, treatment with itraconazole can cost US$26 per day [21]. Thus, there is a clear need for new, effective diagnostics and treatments, accessible to those who need it; simple yet innovative approaches could prove fruitful [5] (for example, staining mycetoma grains with henna for histological analysis[12]).

**Fig 1:**
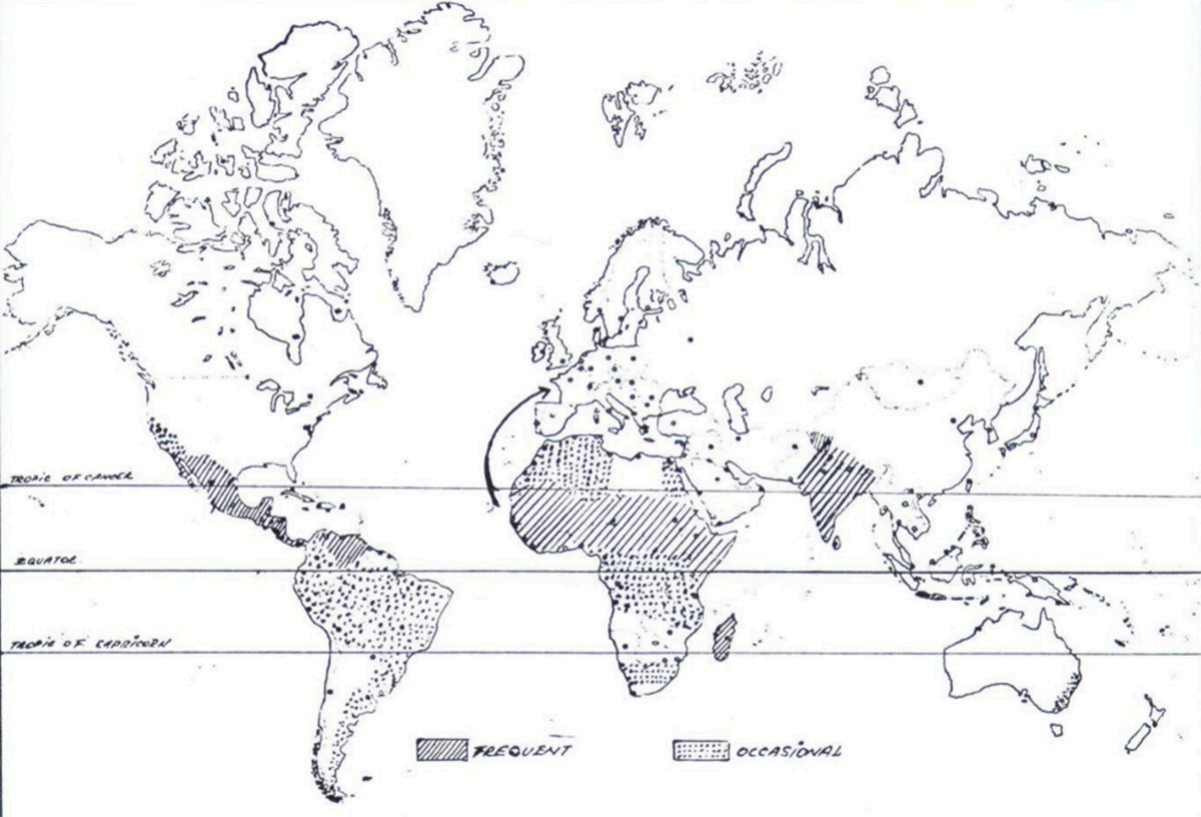
World distribution of mycetoma, 1984 adapted from Magana [24].

Traditionally, the literature talks of a “mycetoma belt” in which most cases of mycetoma are reported. Some authors define it by the countries it encompasses (e.g., “Venezuela, Chad, Ethiopia, India, Mauritania, Mexico, Senegal, Somalia, Sudan, and Yemen”: WHO website) [22], others by its geographical latitude, given as between 30°N and 15°S [23], others still by the wider label of “tropical and subtropical” regions [7]. Clearly, the definition varies between authors, which begins to highlight the inadequacy of epidemiological research on mycetoma; few reports detail its true global distribution. The first such map was produced by Magana in 1984 (Fig 1) [24]; the only one to follow was by van de Sande in 2013 (which was updated in 2018): it included 17,607 cases worldwide [12,13]. Despite the time gap between them, the findings are similar in that Mexico, Senegal, Sudan, and India report most cases, while countries outside the “mycetoma belt” experience a lesser burden. We have updated these maps to present all cases ever reported in the literature and document the ratio of actinomycetoma to eumycetoma.

## Methods

### Search strategy and inclusion criteria

A literature search of Healthcare Databases Advanced Search (HDAS) was carried out, country by country, for case series or case reports from PubMed, Medline, and Cumulative Index to Nursing and Allied Health Literature (CINAHL). Using Boolean operators, the search term for titles and abstracts was “(mycetoma OR “Madura foot”) AND country.” A search of reviews and ongoing trials in each country was conducted using Cochrane Library, with the same terms. The list of countries was obtained from United Nations (UN) International Trade Statistics Country Codes [25], with reference to historic country nomenclature from Nations Online [26]. Countries are organized into geographic regions, in line with UN standard area coding for statistical use [27].

New and old names of organisms were retrieved using PubMed literature searches. Papers in English, French, Italian, and Spanish were included, and automatic translation in Google Chrome was used for other languages, when possible. Their abstracts were read to ascertain relevance: Papers describing “mycetoma” of the internal organs were excluded, as were papers that did not specifically state from which country or time period the data was collected. Finally, this collection of papers was expanded by reference to their secondary sources. Case series were prioritized, with case reports only being used if case series were absent or very small (at the authors’ discretion). In total, 332 papers were included (detailed in Appendix [14]).

### Use of data

The Specialist Unit for Review Evidence (SURE) checklist [28] from Cardiff University was used to assess the reliability and usefulness of each case report and series. For the purposes of this paper, cases were included as “autochthonous,” unless the patient’s nationality was stated as foreign or if the report mentioned migration or travel on the part of the patient (in which case the paper was excluded to avoid duplication). In general, case series from particular countries were presumed to include autochthonous cases only, unless stated otherwise. If a series contained information on a case from another country, this case was not included in either the host or native country’s data.

Once the relevance of a paper was ascertained, it was read to determine the year of study, country of origin, number of cases, type (eumycetoma or actinomycetoma), and species of the predominant causative microorganism. The most common causative organisms for each paper were noted and tallied in a table to determine which organism was reported most frequently in regions (where data was available). When possible, data were used to calculate the percentage ratio of eumycetoma to actinomycetoma. When case series dates overlapped in a country, calculations with average incidence per year (rounded down to avoid duplication) were made to correct the overlap and account for as many cases as possible, in an attempt to compensate for the tendency of mycetoma incidence to be underestimated [7]. When no specified dates were mentioned for data collection, the year of publication was assumed to be the reference year.

After collating the data in a spreadsheet, Quantum Geographic Information System (QGIS) software was used to create the maps. Shapefiles were obtained from Natural Earth [29] (http://www.naturalearthdata.com/about/terms-of-use/).

Using QGIS, the data for the number of cases reported by country were categorized into sets, arranged by the number of cases: “less than 10,” “10–50,” “51–100,” “101–500, and “more than 500.” These categories were chosen to facilitate interpretation because they correlate with previous research into the distribution of mycetoma. Only United Nations–recognized states (as illustrated as the United Nations International Trade Statistics Country Codes) [25] are used in the maps.

## Results

**Fig 2:**
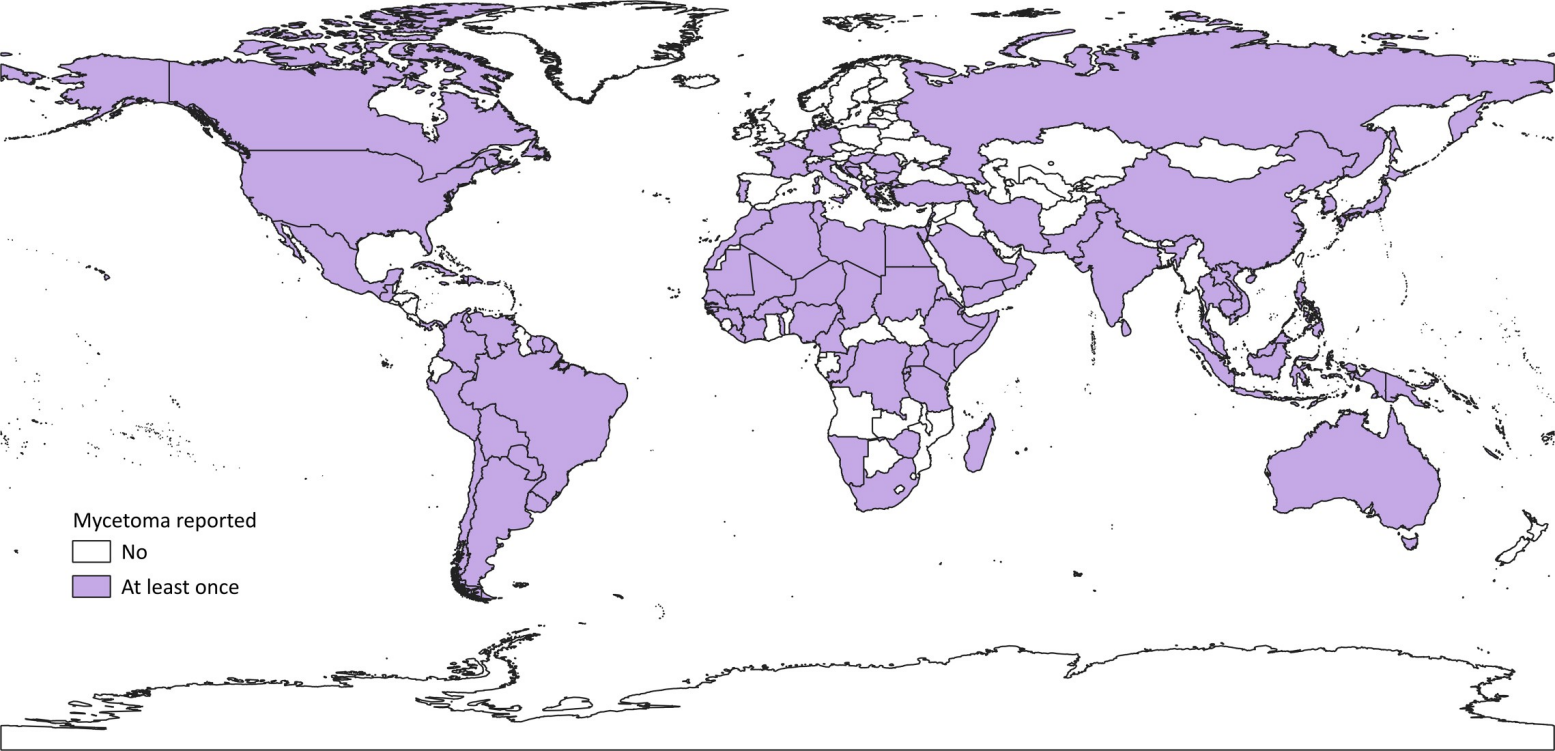
All the countries where autochthonous mycetoma has ever been reported.

In total, 332 papers were included in the data set, numbering 19,494 cases dating from 1876 to 2019 [14]: a higher total than previous papers [12]. Fig 2 details whether or not mycetoma has ever been reported as originating in a given country. To date, according to our literature, mycetoma has been contracted in 102 countries [14], many outside the “mycetoma belt.” The highest number of cases was found in Sudan (10,608), but many countries only reported 1 case: These included Libya, Burundi, Namibia, Antigua and Barbuda, Canada, Vietnam, Russia, Hungary, and Vanuatu [14]. Previously unrepresented countries include the United States (57 cases), Italy (8), China (9) and Australia (5) [14]. With cases reported in North America, Europe, Southern Africa, the Far East, and Australasia, mycetoma is more far-reaching than previously estimated: Reports were found in every continent except Antarctica [14]. There are some notable gaps, in that all countries in sub-Saharan Africa would be expected to have cases given neighboring countries with well-documented cases. Such anomalous countries include Angola, Botswana, Central African Republic, Mozambique, and Zambia. Likewise, we found no cases in Guyana, Ecuador, Nicaragua, Honduras, Syria, Iraq, Turkmenistan, Afghanistan, Nepal, Bangladesh, and Myanmar. It is possible that cases have been reported in local publications, not accessible online, or in the grey literature.

**Fig 3:**
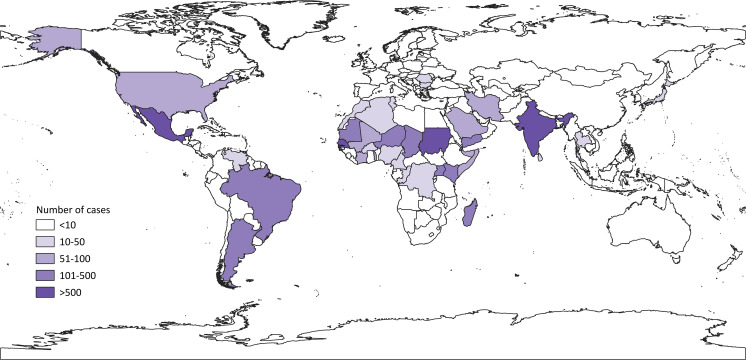
The number of cases of mycetoma reported by country. Darker shades signify more cases.

We next mapped the cumulative number of autochthonous cases that have ever been reported in a country, up to the time of writing (Fig 3). As expected, tropical countries traditionally associated with mycetoma have high numbers of reported cases: Sudan reporting 10,608, Mexico 4,155, and India 1,116 [14]. Countries far outside the tropics report fewer cases: New Zealand reports none, South Africa 10, and Canada only one [14]. Again, gaps emerged: Countries with lots of reports had neighbors with many fewer. For example, Sudan's neighbor Chad reported only 154 cases (representing 1.5% of Sudan’s figure), Guatemala (bordering Mexico) reported two, and Pakistan (bordering India) only one [14].

**Fig 4:**
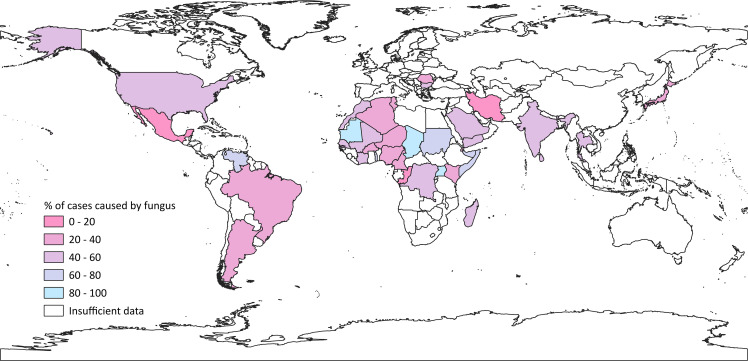
Global map showing the ratio of actinomycetoma to eumycetoma per country, from reported cases. The bluer colors indicate a higher percentage of eumycetoma, while pinker shades indicate more cases caused by bacterial species. White indicates that information was insufficient in the country (either because there were low numbers of reports or because the reports available did not contain adequate species data).

Our third map (Fig 4) illustrates the ratio of eumycetoma to actinomycetoma in each region with more than 10 cases, where blue represents 100% fungal reports and pink represents 100% bacterial. Representation in white signifies insufficient data, either because of low case numbers or no distinction between fungal and bacterial causes. As an example, Togo had two case series: One reported 33 cases of mycetoma (of which 24 were fungal [4]), the other reported 30 cases, without distinction [30]; thus, the proportion of eumycetoma depicted for Togo is 24 of 33 or 73%. Many countries have a roughly equal mix of actinomycetoma and eumycetoma, but some stand out as extremes (for example Iran: 17% eumycetoma, and Chad: 95% eumycetoma) [14]. Areas with predominantly actinomycetoma reports include North and South America and the Middle and Far East. More eumycetoma cases were reported from some central African countries. Mexico, a high-burden country, reported just 3% eumycetoma, Sudan reported 73%, and India reported 42% [14]. As before, discrepancies are seen between neighboring countries, most notably in Africa. For example, Chad reported 95% eumycetoma, while Niger reported only 26%; Mali reported 44%, next to Mauritania with 83% [14]. Some differences may be explained by small sample sizes. Differences are also seen within individual countries: In the neighboring Brazilian states of Rio de Janeiro, Sao Paulo, and Parana, the proportions of fungal mycetoma reported were 62%, 30%, and 33%, respectively [14], although the sample for Brazil was relatively large (235 cases) [14]. This is likely to be an artefact of publication bias, rather than true epidemiological distribution.

Although not visualized in a map, some reports in the literature specified which causative organisms were most common in their investigations, summarized by geographical region in Table 2. Overall, we see that *M*. *mycetomatis* and *Nocardia* spp. are the most common agents of fungal and bacterial mycetoma, respectively.

**Table 2:** Predominant agents of mycetoma, by region [14]. Previous names are shown in brackets, where available.

Region	Predominant agent of eumycetoma	Predominant agent of actinomycetoma
North America	*Scedosporium* spp.	*Nocardia brasiliensis*
Latin America and the Caribbean	*Trematosphaeria grisea (Madurella grisea)*	*N*. *brasiliensis*
South America	*T*. *grisea*	*N*. *brasiliensis*
North Africa	*M*. *mycetomatis (M*. *mycetomi)*	*Actinomadura madurae*
West Africa	*M*. *mycetomatis*	*A*. *pelletieri*
Middle Africa	*Scedosporium* spp.	*Nocardia* spp.
East Africa	*M*. *mycetomatis*	*Nocardia* spp.
West Asia	*M*. *mycetomatis*	*Streptomyces somaliensis*
South Asia	*M*. *mycetomatis*	*A*. *madurae*
South East Asia	*M*. *mycetomatis*	*Nocardia asteroides*

## Discussion

The use of data in these maps is new: It is presented as raw case numbers rather than incidence or prevalence and is, therefore, irrespective of time or population size. With this method, we have gathered 19,494 cases from 102 countries, dating from 1876 to 2019, which equates to roughly 135 cases per year over this 143-year period [14]. While 29 countries (including Libya, Antigua and Barbuda, Canada, Vietnam, Hungary, and Vanuatu) reported only one case, three countries (Mexico, Sudan, and India) reported over 1,000 [14].

In many countries, reports have not been consistent over this time period. For example, several reports were published in the 1980s from Niger, with none reported since; the only two case series from Thailand were published in the 1980s and 1990s; Yemen, source of one of the earliest reports (in 1904), had research published in only a handful of years during the second half of the 20th century; Senegal had a cluster of reports in the 1950s and 1960s and then a decade’s gap before research resumed; in Sudan, the reports dried up during the 1970s to 1990s [14]. Of course, we do not know whether this equates to a real decrease in prevalence, changes in behavior, or something else entirely; in the case of Sudan, gaps in the timeline coincide with its Second Civil War [31]. However, research in Brazil, Mexico, and India has been fairly constant over the decades [14]. Our map contrasts to van de Sande’s 2013 work, in which incidence was shown per year [13]. The gaps in research identified above would dilute these estimates, whereas our use of data is irrespective of time, thus highlighting the burden of countries like Chad (reporting 153 cases over two studies, nearly 40 years apart) [14].

The binary data for each country (Fig 2) shows that mycetoma has been reported in every populated continent on earth. This represents a larger distribution than previous maps, because previous maps were based on incidence and prevalence and so would not include countries with very few cases per population or time period. Fig 2 shows that mycetoma is so widespread that some gaps become apparent for countries whose neighbors report cases, notably in sub-Saharan Africa and Latin America. Like Fig 2, the second map (Fig 3) also highlights gaps in mycetoma’s distribution where neighboring countries would be expected to have similar case numbers. As we have seen, 29 countries reported only one case and some of these (for example, Libya) neighbor countries with well-documented cases. In these areas, the burden of mycetoma is surely larger. As other authors note [12], basic epidemiological study of mycetoma is often absent.

Fig 3 portrays the global distribution of the 19,494 cases of mycetoma. This is a larger figure than previous studies on the topic: the last (van de Sande, 2018) collated 17,607 cases [12]. There may be several reasons for this. Firstly, our search was more recent: 20 countries reported cases in the interim, including some (Eritrea, Hungary, Timor Leste) for the first time [14]. Furthermore, unlike van de Sande’s prevalence map, Fig 3 does not take population size into account. Thus it presents a wider distribution, with representation for countries like Kenya whose 155 cases [14] were previously hidden by its population size. In our search strategy, more databases were used and more languages included. In endemic countries, under-reporting is likely, and resources necessary to accurately diagnose mycetoma may not be available [12]. Even in Sudan, home to the only Mycetoma Research Centre (MRC) in the world, the 10,608 reported cases [14] are likely to be an underestimate [13]: While van de Sande’s 2013 study calculated prevalence as 1.81 per 100,000 population (most cases coming from the MRC located in the capital Khartoum), a study in rural Sudan found it to be as high as 6.2 per 1,000 [13]. As mycetoma is associated with rural agricultural practices [8] and use of local herbal remedies [12], it may be that many cases do not present to large healthcare facilities, from where reports are likely to be published.

Our third map shows the ratio of actinomycetoma to eumycetoma (Fig 4) in countries with more than 10 cases, where data was available. This map shows that, for many countries, mycetoma is caused by a mix of fungal and bacterial organisms. Only a few countries stand out as having a high proportion caused by either fungi or bacteria; such extreme examples include Mauritania (83% fungal), Chad (95%), Mexico (3%), and Iran (17%) [14]. Overall, actinomycetoma is seen more in the Americas (in keeping with previous research) [13] and the Middle and Far East. Areas with majority eumycetoma cases included some central African countries. Once again, stark differences can be seen on the map between neighboring countries, and the literature revealed differences even within countries themselves (although this is not represented in our maps). In some cases, it may be a result of small sample size (for example, in Jamaica, 84% of 19 cases were fungal) [14]; in others, publication bias may affect the likelihood of researchers reporting a particular type of mycetoma (as was the case in the 1995 study by Venugopal and Venugopal which only included fungal mycetomas in India, where bacterial forms are more common) [32]. We cannot be certain why these differences arise (whether they are real epidemiological findings, or a product of diagnostic or publication bias) without further research.

The most common causative agents found in each region are presented in Table 2. Globally, this shows a predominance of *M*. *mycetomatis* and *Nocardia* spp. In van de Sande’s study, *M*. *mycetomatis* was found to be the most common agent of all cases, fungal or otherwise; *Nocardia* spp. were included only as one of a group of most common agents of actinomycetoma–alongside *A*. *madurae*, *S*. *somaliensis*, *A*. *pelletieri* [13]. Our use of species data was qualitative, while van de Sande analyzed this quantitively [13].

Samples were mostly studied by histology, which is inaccurate in distinguishing between species [13]. Indeed, subsequent study of some samples revealed that old isolates, classed as containing one species, actually contained more than one; species thought to be genetically related have also emerged as different genera [33]. In Table 1 are some rare causative agents of mycetoma. Some, like *Curvularia lunata*, were only reported once [14]; in light of the inaccuracies shown with other diagnoses, their rarity may indicate erroneous identification. The lack of grains in mycetoma caused by *Scedosporium spp* [34] points to inaccuracies in diagnosis. In the absence of an objective diagnostic tool, attempts to obtain accurate results in any epidemiological study are hindered.

As Figs 2 and 3 show, the reach of mycetoma has expanded beyond its traditional tropical endemic areas. Autochthonous cases were published from as far north as Canada, Russia, Germany, and Japan. It is conceivable that globalization in the form of increased migration, tourism, and trade may contribute to the spread of mycetoma from its endemic areas [35]. Occupational exposure to cotton from endemic regions could be a route of infection, as in a case from the United Kingdom [36]. Alongside globalization, climate change could affect the distribution of mycetoma, as for other infectious diseases like malaria [37,38].

One nontropical autochthonous mycetoma was found in an immunocompromised patient: a transplant recipient in the United States [39], the only published example. In the immunocompromised, causative species such as *Nocardia* are more likely to cause invasive infections like pneumonia [40]. Rarely has mycetoma been reported in patients living with HIV [41]. However, many gaps remain in the epidemiological study of mycetoma [12], so it is difficult to ascertain whether these nontropical outliers are simply anomalous or part of a trend.

Although our maps show that mycetoma is more widespread than previously estimated, the literature also reveals major inconsistencies in research on its epidemiology. Two recent publications highlight this. Firstly, from 2016 to 2017, WHO conducted a global survey on mycetoma [42]. Sending questionnaires to all WHO regions (except Europe), regional offices were asked about mycetoma prevalence, surveillance, research, and treatment protocols. The outcome was disappointing: Alongside a poor response rate, 25% of respondents did not know whether mycetoma had ever been reported in their country [42]. In addition, there were inconsistencies in treatment, and only Jordan had official guidelines for management (even though no cases were reported in the WHO survey or ours) [14,42]. Furthermore, this WHO survey revealed that El Salvador had 53 cases between 2014 and 2016 [42]. However, zero cases appeared in our literature search, despite Spanish papers from El Salvador being actively sought (see Methods), suggesting that these reports were published in sources not available through our databases. This significant discrepancy in case numbers highlights the need for international collaboration on mycetoma research in order to increase its visibility.

Another recent paper by Kwizera and colleagues on mycetoma in Uganda [43] emphasizes the propensity of this NTD to be misdiagnosed. In our original literature review, only 30 cases were identified for Uganda, from only two case series published in 1958 [44] and 1965 [45]. However, when Kwizera and colleagues reviewed the biopsy reports of the Pathology Department at Makerere University from 1950 to 2019, they found an additional 249 cases; the authors cite a low index of suspicion for mycetoma amongst clinicians as a reason for this [43]. The perceived rarity of mycetoma compounded underdiagnosis. Similarly, the “mycetoma belt” has traditionally represented a geographical proxy for where mycetoma is likely to be found. However, its definition is disputed in the literature, and, as we have seen, mycetoma has actually been found in countries all over the world. Thus, its use may foster complacency and ignorance about mycetoma in countries not associated with the label, leading to underdiagnosis. This self-perpetuating cycle of underdiagnosis and subsequent under-estimation will continue until the true burden is ascertained.

We recognize many limitations in a literature review of this kind, especially publication bias. This will lead to inaccurate estimates of prevalence, as academic interest in mycetoma will differ between countries and therefore affect likelihood of reporting, positively or negatively. For example, under-reporting in endemic countries, such as India, is expected. Missing data in the literature are common: dates of study not specified, patient’s place of residence not noted, cases from more than one country described (without distinction between them), and data reported from historic countries (which now have different boundaries). In these cases, the data could not be included in the analysis.

During analysis, some features of the research made it difficult to ensure accurate use of the data. In some older papers, the type of mycetoma was not mentioned. Later the color of grains was described but not whether it was fungal or bacterial. Inferring the type of mycetoma by color (with reference to a 2004 paper by Ahmed and colleagues [16] will not always be accurate, as it relies on subjective descriptions from very old papers. However, as the practice of determining type by grain color was so ubiquitous in the literature, it seemed appropriate that these reports were included, despite likely unreliability in ratios between eumycetoma and actinomycetoma. Even if the distinction was made between fungal and bacterial forms, papers without detail of causative species were common. Indeed, in many regions where mycetoma is prevalent, the resources necessary to identify the species of the causative organism are simply not available [12].

For the purposes of analysis, it was assumed that every case in the literature was independent if reports did not overlap in time or national boundary. Occasional duplication of cases is likely.

In spite of these limitations, our search has revealed a higher number of mycetoma reports in more countries than ever and highlighted persistent gaps in knowledge. This should fuel more in-depth research into mycetoma everywhere and encourage increased awareness among healthcare professionals. These maps may have significant potential: Reference can be made to whether mycetoma has ever been reported in a country, how common it has been, and which type is likely to occur. This should not only influence local lay knowledge and clinical decision-making but also public health initiatives, diagnostic development and sustainable supply chains for necessary treatments. However, until the inconsistencies in epidemiological research on mycetoma are addressed, these uses cannot be implemented.

### Conclusions

Concentrated in (but not limited to) the tropics, mycetoma is a potentially debilitating skin disease. Its chronicity, many diverse causative organisms, and lack of an objective diagnostic tool make epidemiological study difficult. Our maps have shown mycetoma to be more widespread than previously represented in the literature. In a world of globalization and climate change, autochthonous cases are emerging in previously untouched regions. Differences in reporting show that countries need to work together to further this effort. These maps have the capacity to shape the management of mycetoma at all levels but epidemiological granularity is required.

## Supporting information

S1 Appendix Papers used in maps, by region. Emery D, Denning DW. The global distribution of actinomycetoma and eumycetoma. *Regions as per United Nations standard area codes for statistical use*.
(DOCX)

## References

[1] Javed F, Nazir R, Sharma M, Yasir S, Babar S. Dot-in-Circle Sign—A Diagnostic MRI Sign for “Madura Foot”. J Coll Physicians Surg Pak [Internet]. 2017 3 [cited 2019 Oct 21];27(3):S8–10. Available from: http://www.ncbi.nlm.nih.gov/pubmed/28302229 doi: 236 28302229

[2] Estrada R, Chávez-López G, Estrada-Chávez G, López-Martínez R, Welsh O. Eumycetoma. Clin Dermatol [Internet]. 2012 [cited 2019 Oct 21];30(4):389–96. Available from: http://www.ncbi.nlm.nih.gov/pubmed/22682186 10.1016/j.clindermatol.2011.09.009 22682186

[3] Salim AO, Mwita CC, Gwer S. Treatment of Madura foot: A systematic review. Vol. 16, JBI Database of Systematic Reviews and Implementation Reports. Joanna Briggs Institute; 2018 p. 1519–36. 10.11124/JBISRIR-2017-003433 29995713

[4] Darré T, Saka B, Mouhari-Toure A, Tchaou M, Dorkenoo AM, Doh K, et al Mycetoma in the togolese: An update from a single-center experience. Mycopathologia. 2018 3 20;183(6):961–5. 10.1007/s11046-018-0260-y 29557534 PMC6305724

[5] van de Sande WWJ, Maghoub ES, Fahal AH, Goodfellow M, Welsh O, Zijlstra E. The Mycetoma Knowledge Gap: Identification of Research Priorities. PLoS Negl Trop Dis. 2014;8(3). 10.1371/journal.pntd.0002667 PMC3967943 24675533

[6] Develoux M. [Mycetoma and their treatment]. J Mycol Med [Internet]. 2016 6 [cited 2019 Oct 21];26(2):77–85. Available from: http://www.ncbi.nlm.nih.gov/pubmed/27260344 10.1016/j.mycmed.2016.03.005 27260344

[7] Samy AM, van de Sande WWJ, Fahal AH, Peterson AT. Mapping the Potential Risk of Mycetoma Infection in Sudan and South Sudan Using Ecological Niche Modeling. PLoS Negl Trop Dis. 2014;8(10). 10.1371/journal.pntd.0003250 PMC4199553 25330098

[8] Reis CMS, Reis-Filho EG de M. Mycetomas: An epidemiological, etiological, clinical, laboratory and therapeutic review. Vol. 93, Anais Brasileiros de Dermatologia. Sociedade Brasileira de Dermatologia; 2018 p. 8–18. 10.1590/abd1806-4841.20187075 PMC5871356 29641691

[9] Abbas M, Scolding PS, Yosif AA, EL Rahman RF, EL-Amin MO, Elbashir MK, et al The disabling consequences of Mycetoma. PLoS Negl Trop Dis. 2018 12 1;12(12). 10.1371/journal.pntd.0007019 PMC6312340 30532253

[10] Nenoff P, van de Sande WWJ, Fahal AH, Reinel D, Schöfer H. Eumycetoma and actinomycetoma—an update on causative agents, epidemiology, pathogenesis, diagnostics and therapy. J Eur Acad Dermatol Venereol [Internet]. 2015 10 [cited 2019 Oct 21];29(10):1873–83. Available from: http://www.ncbi.nlm.nih.gov/pubmed/25726758 10.1111/jdv.13008 25726758

[11] Mycetoma is added to WHO List of ‘Neglected Tropical Diseases’–DNDi [Internet]. [cited 2020 Apr 4]. Available from: https://www.dndi.org/2016/media-centre/press-releases/mycetoma-who-ntd-list-response/

[12] Van De Sande W, Fahal A, Ahmed SA, Serrano JA, Bonifaz A, Zijlstra E. Closing the mycetoma knowledge gap. Med Mycol. 2018;56:S153–64. 10.1093/mmy/myx061 28992217

[13] van de Sande WWJ. Global Burden of Human Mycetoma: A Systematic Review and Meta-analysis. PLoS Negl Trop Dis. 2013;7(11). 10.1371/journal.pntd.0002550 PMC3820768 24244780

[14] Emery D. Appendix. 2020.

[15] Maiti PK, Ray A, Bandyopadhyay S. Epidemiological aspects of mycetoma from a retrospective study of 264 cases in West Bengal. Trop Med Int Heal. 2002;7(9):788–92. 10.1046/j.1365-3156.2002.00915.x 12225511

[16] Ahmed AOA, Van Leeuwen W, Fahal A, Van De Sande W, Verbrugh H, Van Belkum A. Mycetoma caused by Madurella mycetomatis: A neglected infectious burden. Vol. 4, Lancet Infectious Diseases. 2004 p. 566–74. 10.1016/S1473-3099(04)01131-4 15336224

[17] Zijlstra EE, van de Sande WWJ, Welsh O, Mahgoub ES, Goodfellow M, Fahal AH. Mycetoma: a unique neglected tropical disease. Lancet Infect Dis [Internet]. 2016 1 [cited 2019 Oct 21];16(1):100–12. Available from: http://www.ncbi.nlm.nih.gov/pubmed/26738840 10.1016/S1473-3099(15)00359-X 26738840

[18] Gismalla MDA, Ahmed GMA, Mohamedali MM, Taha SM, Mohamed TA, Ahmed AE, et al Surgical management of eumycetoma: Experience from Gezira Mycetoma Center, Sudan 11 Medical and Health Sciences 1103 Clinical Sciences. Trop Med Health [Internet]. 2019 1 14 [cited 2020 Apr 15];47(1):6 Available from: https://tropmedhealth.biomedcentral.com/articles/10.1186/s41182-018-0129-2 30675125 10.1186/s41182-018-0129-2 PMC6332587

[19] Fahal AH. Mycetoma Management Guidelines [Internet]. [cited 2019 May 3]. Available from: http://www.mycetoma.edu.sd/index.php/mycetoma-management-guidelines

[20] WHO. WHO Model List of Essential Medicines [Internet]. 2017 [cited 2019 Jun 6]. Available from: http://www.who.int/medicines/publications/essentialmedicines/en/

[21] Bakhiet SM, Fahal AH, Musa AM, Mohamed ESW, Omer RF, Ahmed ES, et al A holistic approach to the mycetoma management. PLoS Negl Trop Dis. 2018 5 10;12(5). 10.1371/journal.pntd.0006391 PMC5944909 29746460

[22] WHO | Mycetoma [Internet]. [cited 2020 Apr 14]. Available from: https://www.who.int/buruli/mycetoma/en/

[23] Relhan V, Mahajan K, Agarwal P, Garg VK. Mycetoma: An update Vol. 62, Indian Journal of Dermatology. Medknow Publications; 2017 p. 332–40. 10.4103/ijd.IJD_476_16 28794542 PMC5527712

[24] Magana M. Mycetoma. Int J Dermatol [Internet]. 1984 5 [cited 2019 Oct 21];23(4):221–36. Available from: 10.1111/j.1365-4362.1984.tb01238.x 6376380

[25] Country Code (ISO 3) [Internet]. [cited 2019 Oct 21]. Available from: https://unstats.un.org/unsd/tradekb/knowledgebase/country-code

[26] Historical Country Names—Nations Online Project [Internet]. [cited 2019 Apr 11]. Available from: https://www.nationsonline.org/oneworld/historical_countrynames.htm

[27] UNSD—Methodology [Internet]. [cited 2020 Apr 15]. Available from: https://unstats.un.org/unsd/methodology/m49/

[28] Specialist Unit for Review Evidence (SURE). [cited 2019 Apr 11]; Available from: http://www.cardiff.ac.uk/insrv/libraries/sure/checklists.html

[29] 10m-cultural-vectors | Natural Earth [Internet]. [cited 2019 May 27]. Available from: https://www.naturalearthdata.com/downloads/10m-cultural-vectors/

[30] Pitche P, Napo-Koura G, Kpodzro K, Tchangai-Wallam K. Les mycétomes au Togo: Aspects épidémiologiques et étiologiques des cas histologiquement diagnostiqués. Med Afr Noire. 1999;46(6):322–5.

[31] Second Sudanese Civil War (1983–2005) [Internet]. [cited 2020 May 1]. Available from: https://www.blackpast.org/global-african-history/second-sudanese-civil-war-1983-2005/

[32] Venugopal P V., Venugopal T V. Pale grain eumycetomas in Madras. Australas J Dermatol. 1995;36(3):149–51. 10.1111/j.1440-0960.1995.tb00957.x 7487741

[33] Borman AM, Desnos-Ollivier M, Campbell CK, Bridge PD, Dannaoui E, Johnson EM. Novel taxa associated with human fungal black-grain mycetomas: Emarellia grisea gen. nov., sp. nov., and Emarellia paragrisea sp. nov. J Clin Microbiol. 2016;54(7):1738–45. 10.1128/JCM.00477-16 27076666 PMC4922095

[34] Posteraro P, Frances C, Didona B. Persistent subcutaneous Scedosporium apiospermum infection. Eur J Dermatol. 2003;13:603–5. 14721787

[35] Abraham BJ, Km D. An unusual presentation of eumycetoma mimicking soft tissue sarcoma of foot [Internet]. Vol. 26, Kerala Journal of Orthopaedics. 2013 [cited 2020 May 1]. Available from: www.kjoonline.orgwww.kjoonline.orgQuickresponsecode

[36] Cerar D, Malallah YM, Howard SJ, Bowyer P, Denning DW. Isolation, identification and susceptibility of Pyrenochaeta romeroi in a case of eumycetoma of the foot in the UK. Int J Antimicrob Agents [Internet]. 2009 12 [cited 2019 Oct 21];34(6):617–8. Available from: http://www.ncbi.nlm.nih.gov/pubmed/19783408 10.1016/j.ijantimicag.2009.08.004 19783408

[37] Lafferty KD. The Ecology of Climate Change and Infectious Diseases. Vol. 90, Source: Ecology. 2009. 10.1890/08-0079.1 19449681

[38] Endo N, Eltahir EAB. Increased risk of malaria transmission with warming temperature in the Ethiopian Highlands Increased risk of malaria transmission with warming temperature in the Ethiopian Highlands. Environ Res Lett. 2020;15(054006).

[39] Hopps S, Roach A, Yuen C, Borders E. Treatment for a eumycetoma infection caused by Aspergillus in an immunocompromised host: A case report. Transpl Infect Dis. 2015 2 1;17(1):94–7. 10.1111/tid.12321 25537527

[40] Sharma NL, Mahajan VK, Agarwal S, Katoch VM, Das R, Kashyap M, et al Nocardial mycetoma: diverse clinical presentations. Indian J Dermatol Venereol Leprol [Internet]. 2017 [cited 2019 Oct 21];74(6):635–40. Available from: http://www.ncbi.nlm.nih.gov/pubmed/19171991 10.4103/0378-6323.45110 19171991

[41] Castro LG, Valente NY, Germano JA, Vaccari EM, da Silva Lacaz C. Mycetoma in an HIV-infected patient. Rev Hosp Clin Fac Med Sao Paulo. 1999;54(5):169–71. 10.1590/s0041-87811999000500008 10788840

[42] World Health Organisation, WHO. Weekly epidemiological record: Results of the 2017 global WHO survey on mycetoma [Internet]. Vol. 33, WHO epidemiological record. 2018 Aug [cited 2020 Apr 4]. Available from: http://www.who.int/wer2018,93,417-428No33

[43] Kwizera R, Bongomin F, Meya DB, Denning DW, Fahal AH, Lukande R. Mycetoma in Uganda: A neglected tropical disease. Poonawala H, editor. PLoS Negl Trop Dis [Internet]. 2020 Apr 29 [cited 2020 May 1];14(4):e0008240 Available from: https://dx.plos.org/10.1371/journal.pntd.0008240 32348300 10.1371/journal.pntd.0008240 PMC7190103

[44] Davies AG. The bone changes of Madura foot; observations on Uganda Africans. Radiology. 1958 6 1;70(6):841–7. 10.1148/70.6.841 13554851

[45] Wilson A. The Aetiology of Mycetoma in Uganda Compared with Other African Countries. East Afr Med J. 1965;42(5):182–90. 14342777

