# Supplementary material for: The global distribution of actinomycetoma and eumycetoma
Source: PLoS Negl Trop Dis. 2020 Sep 24;14(9):e0008397. doi: 10.1371/journal.pntd.0008397 (PMC7514014; doi:10.1371/journal.pntd.0008397)
Supplement: S1 Appendix — Emery D, Denning DW. The global distribution of actinomycetoma and eumycetoma. Regions as per United Nations standard area codes for statistical use. (DOCX) [file pntd.0008397.s001.docx]

**S1 Appendix: papers used in maps, by region**

Emery et al. The global distribution of actinomycetoma and eumycetoma

**EASTERN AFRICA**

**Burundi**

- Izarn R, Reguer M, ‘A case of actinomycetes mycetoma in Burundi’, Bull Soc Pathol Exot Filiales. 1984, 77(5), 649-652.

**Comoros**

- Epelboin L, Woessner J, Roussin C et al, ‘Actinomycetoma of the knee due to Nocardia otitidiscaviarum described in the Comoro Islands’, Ann Dermatol Venereol. 2013, 140(4), 287-290.

**Djibouti**

- Bourrel P, Andreu JM, Cazenave JC, ‘Mycétomes de la main’, Ann Chir. 1989, 43(10), 814-823.
- Callebaut G, Hooghe L, Dratwa M. ‘Madura's foot in a renal transplant patient: report of a case’, NDT Plus. 2011, 4(6), 397–398. doi:10.1093/ndtplus/sfr109
- Carteron B, Bruneau M, Morvan D et al, ‘Human mycoses in the Republic of Djibouti’, Bull Soc Pathol Exot Filiales. 1978, 71(1), 63-70.
- Orio J, Destombes P, Mariat F et al, ‘Les mycétomes en Côte Française des Somalis’, Bull Soc Pathol Exot Filiales. 1963, 56(2), 161-173.

**Eritrea**

- Mekoguem C, Triboulet C, Gouveia A, ‘Madurella mycetomatis infection of the buttock in an Eritrean refugee in Switzerland: a case report’, J Med Case Rep. 2019, 13(1), 32.
- Schibli A, Goldenberger D, Krieg A et al, ‘Painless swelling of the forefoot and recurrent subcutaneous abscesses of the lower leg—Two distinct presentations illustrating the spectrum of eumycetoma in a nonendemic country’, PLoS Negl Trop Dis. 2017, 11(4), available from: doi.org/10.1371/journal.pntd.0005360

**Ethiopia**

- Destombes P, Mariat F, Rosati L et al, ‘Épidémiologie- Les mycétomes en République de Somalie’, C R Acad Sci Hebd Seances Acad Sci D. 1966, 263(25), 2026-2064.
- Fielder JF, Otieno T, Solfelt D. ‘Case 5--a 38-year-old man presented to Kijabe Mission Hospital with a swollen, draining right foot.’ MedGenMed. 2004, 6(2), 55. Available at <https://www.ncbi.nlm.nih.gov/pmc/articles/PMC1395769/>

**Kenya**

- Cameron HM, Gatei D, Bremner AD, ‘The deep mycoses in Kenya: A histopathological study. 1. Mycetoma’, East Afr Med J. 1973, 50(8), 382-395.
- Maina AM, Macharia JT, ‘Alleviating a Nomad's Anguish: Successful Treatment of a Case of Leg Mycetoma—A Case Report’, Case Rep Orthop. 2012, ID 753174. Available from<https://doi.org/10.1155/2012/753174>

**Madagascar**

- Coulanges P, Vicens R, Rakotonirina-Randriambeloma PJ, ‘Les mycetomas à Madagascar’, Arch Inst Pasteur Madagascar. 1987, 53(1), 35-42.

**Rwanda**

- Vanderick F, Spanoghe L, Dockx P, ‘Mycetomas due to Nocardia observed in Rwanda’, Ann Soc Belg Med Trop. 1974, 54(3), 159-65.

**Somalia**

- Destombes P, Mariat F, Rosati L et al, ‘Épidémiologie- Les mycétomes en République de Somalie’, C R Acad Sci Hebd Seances Acad Sci D. 1966, 263(25), 2026-2064.
- El Muttardi N, Kulendren D, Jemec B, ‘Madura foot - mind the soil’, J Plast Reconstr Aesthet Surg. 2010, 63(7), 576-578. Available at doi: 10.1016/j.bjps.2009.12.007

**Uganda**

- Davies AGM, ‘The Bone Changes of Madura Foot’, Radiology. 1958, 70(6), 841-847.
- Wilson AMM, ‘The Aetiology of Mycetoma in Uganda compared with other African Countries’, East Afr Med J. 1965, 42(5), 182-190. Available from cabdirect.org: 19662900713

**Tanzania**

- Gosselink C, Thomas J, Brahmbhatt S et al, ‘Nocardiosis causing pedal actinomycetoma: a case report and review of the literature’, J Foot Ankle Surg. 2008, 47(5), 457-462. Available from https://www.sciencedirect.com/science/article/pii/S1067251608001439

**Zimbabwe**

- Freland C, Fur JL, Nemirovsky-Trebucq B et al, ‘Primary cutaneous nocardiosis caused by Nocardia otitidiscaviarum: two cases and a review of the literature’, J Trop Med Hyg. 1995, 98(6), 395-403.

**MIDDLE AFRICA**

**Cameroon**

- Destombes P, ‘Deep mycoses identified in 9 years’ histopathological practice in the Pasteur Institute, Cameroun’, Bull Soc Path Ex. 1970, 63(3), 310-315.
- Gamet A, ‘New cases of mycetoma detected in Cameroun’, Bull Soc Path Ex. 1964, 57(6), 1191-1195.
- Mendouga Menye CRB, Kouotou EA, Atangana PJA, ‘Mycétome : apport de l’histopathologie au diagnostic chez un commerçant camerounais, et possibilité d’une contamination urbaine’, J Mycol Med. 2017, 27(3), 417-420.

**Chad**

- Destombes P, ‘Summary of deep mycoses identified in 9 years’ histopathological practice in the Pasteur Institute, Cameroun’, Bull Soc Path Ex. 1970, 63(3), 315-324.
- Ellabib MS, Refaai A, Khalifa Z et al, ‘Isolation and identification of Madurella mycetomatis from two cases of black grain mycetoma in Libya’, Mycoses. 2003, 46(8), 339-41. Available from<https://www.ncbi.nlm.nih.gov/pubmed/12950906>

**Congo**

- Courtois C, Doupagne P, Thys A et al, ‘Two cases of Congo mycetoma caused by Nocardia brasiliensis’, Ann Soc Belg Med Trop. 1956, 36(4), 479-85.
- Destombes P, ‘Summary of deep mycoses identified in 9 years’ histopathological practice in the Pasteur Institute, Cameroun’, Bull Soc Path Ex. 1970, 63(3), 315-324.
- Thys, A, ‘Personal observations on the histopathology of Congolese mycoses’, Ann Soc Belges Med Trop Parasitol Mycol. 1964, 44, 887-895.

**Democratic Republic of the Congo**

- Courtois G, De Loof C, Thys A, et al, ‘Neuf cas de pied de madura congolais’, Ann Soc Belg Med Trop. 1954, 34(4), 371-395.
- Vanbreuseghem R, ‘Mycetoma of the nape of the neck in a Negro of the Belgian Congo’, Ann Soc Belg Med Trop. 1959, 39, 227-238.
- Vanbreuseghem R, Courtois C, Thys A et al, ‘Deux cas de myceétomes congolais par Nocardia brasiliensis’, Ann Soc Belg Med Trop. 1956, 36(4), 479-85.
- Vandepitte J, Beeckmans G, Ninane J, ‘The first case of Madura foot caused by Madurella grisea in the Belgian Congo’, Ann Soc Belg Med Trop. 1956, 36(4), 493-497. Available at cabdirect.org: 19591302345

**NORTHERN AFRICA**

**Algeria**

- Destombes P, ‘Mycetoma due to Streptomyces somaliensis seen in Algeria south of Atlas’, Bull Soc Path Ex. 1965, 58(6), 1017-1020.

**Egypt**

- Abdei-Aal H, ‘Madurella mycetomi as a cause of maduromycosis in Egypt’, Dermatol Monatsschr. 1974, 160(8), 670-674.
- El Dine Massoud G, ‘The radiological picture of mycetoma’, Egypt J Intern Med. 1953, 32(1), 64-68.
- El-Mofty AM, Iskander IO, Nada M et al, ‘“Madura foot” in Egypt’, Br J Dermatol. 1965, 77, 365-372.

**Libya**

- Ellabib MS, Refaai A, Khalifa Z et al, ‘Isolation and identification of Madurella mycetomatis from two cases of black grain mycetoma in Libya’, Mycoses. 2003, 46(8), 339-341.

**Morocco**

- Amrani FE, Hassam B, ‘Le pied de Madura’, Pan Afr Med J. 2013. Available at http://www.panafrican-med-journal.com/content/article/14/24/full/
- Asly M, Rafaoui A, Bouyermane H et al, ‘Mycetoma (Madura foot): A case report’, Ann Phys Rehabil Med. 2010, 53(10), 650-654.
- Baha H, Khadir K, Hali F et al, ‘Mycétome actinomycosique du pied à Actinomycetes viscosus au Maroc’, J Mycol Med. 2015, 25(1), 76-80.
- Bouhamidi A, Boui M, ‘Madura foot: a case report’, Pan Afr Med J. 2018. Available at http://www.panafrican-med-journal.com/content/article/30/131/pdf/131.pdf
- Efared B, Tahiri L, Boubacar MS et al, ‘Mycetoma in a non-endemic area: a diagnostic challenge’, BMC Clin Pathol. 2017, 17(1), 1-6. Available at <https://bmcclinpathol.biomedcentral.com/track/pdf/10.1186/s12907-017-0040-5>
- Marc S, Meziane M, Hamada S, ‘Clinique et épidémiologie des mycétomes au Maroc’, Med Mal Infect. 2011, 41(3), 163-164. Available at doi 10.1016/j.medmal.2010.11.006
- Messoudi A, Fnini S, El Andaloussi Y et al, ‘Le pied de Madura: pathologie rare au Maroc (à propos de 15 cas)’, Bull Soc Path Ex. 2013, 106(9), 9-12.

**Sudan**

- Abbas M, Scolding PS, Yosif AA et al, ‘The disabling consequences of Mycetoma’, PLoS Negl Trop Dis. 2018, 12(12), 1-16. Available at https://doi.org/10.1371/journal.pntd.0007019
- Abbott P, ‘Mycetoma in the Sudan’, Trans R Soc Trop Med Hyg. 1956, 50(1), 11–30.
- Aounallah A, Boussofara L, Ben Saïd Z et al, ‘Analyse d’une série tunisienne de 18 cas de mycétomes à l’hôpital de Sousse (1974–2010)’, Bull Soc Path Ex. 2013, 106(5), 5-8.
- El Hassan AM, Mahgoub ES, ‘Lymph node involvement in mycetoma’, Trans R Soc Trop Med Hyg. 1972, 66(1), 165-169.
- El-Bagi A, Fahal AH, ‘Mycetoma revisited’, Saudi Med J. 2009, 30(4), 529-533. Available at https://www.smj.org.sa/index.php/smj/article/view/6587/4361
- Ezaldeen EA, Fahal AH, Osman A, ‘Mycetoma Herbal Treatment: The Mycetoma Research Centre, Sudan Experience’, PLoS Negl Trop Dis. 2013, 7(8), 1-5. Available at https://doi.org/10.1371/journal.pntd.0002400
- Fahal A, Mahgoub el S, El Hassan AM et al, ‘Mycetoma in the Sudan: an update from the Mycetoma Research Centre, University of Khartoum, Sudan’, PLoS Negl Trop Dis. 2015, 9(3). Available at doi:10.1371/journal.pntd.0003679
- Gismalla MDA, Ahmed GMA, MohamedAli MM et al, ‘Surgical management of eumycetoma: experience from Gezira Mycetoma Center, Sudan’, Trop Med Int Health. 2019, 47(6), 1-6. Available at <https://tropmedhealth.biomedcentral.com/track/pdf/10.1186/s41182-018-0129-2>
- Lynch JB, ‘Mycetoma in the Sudan’, Ann R Coll Surg Engl. 1964, 35(6), 319–340. Available at https://www.ncbi.nlm.nih.gov/pmc/articles/PMC2311794/
- Mahgoub ES, ‘Medical management of mycetoma’, Bull World Health Organ. 1976, 54(3), 303–310.
- Wadal A, Elhassan TA, Zein HA et al, ‘Predictors of Post-operative Mycetoma Recurrence Using Machine-Learning Algorithms: The Mycetoma Research Center Experience’,  PLoS Negl Trop Dis. 2016. Available at <https://doi.org/10.1371/journal.pntd.0005007>
- Zein HAM, Fahal AH, Mahgoub ES et al, ‘Predictors of cure, amputation and follow-up dropout among patients with mycetoma seen at the Mycetoma Research Centre, University of Khartoum, Sudan’, Trans R Soc Trop Med Hyg. 2012, 106(11), 639-644.

**SOUTHERN AFRICA**

**Namibia**

- Ide L, Knol J, De Beeck LO et al, ‘Subcutaneous nodules, some 20 years after a fall in Namibia, diagnosed in Belgium: imported pathology may take a long time before diagnosis’, J Clin Pathol. 2009, 62, 765.

**South Africa**

- Arif M, ‘Madura foot’, S Afr Med J. 2007, 97(9), 834-835.
- Corr P, ‘Clinica in diagnostic imaging’, Singapore Med J. 1997, 38(6), 268-269.
- Dlova NC, Mosam A, ‘Actinomycetoma’, Skinmed. 2012, 10(2), 98-100.
- Dlova NC, Mosam A,’Mycetoma’, Skinmed. 2012, 10(3), 181-182.
- Freed CC, Bissett E, Martin PM et al, ‘Actinomycotic mycetoma due to Streptomyces somaliensis: report of a case in South Africa’, Sabouraudia. 1975,13(3), 316-22.
- Motswaledi HM, Mathekga K, Sein PP et al, ‘Paecilomyces lilacinus eumycetoma’, Int J Dermatol. 2009, 48, 858-861.
- Polden KE, Jehle H. ‘Actinomycosis of the foot - A South African case’, S Afr J Surg.  2017, 55(2), 36-37. Available from: <http://www.scielo.org.za/scielo.php?script=sci_arttext&pid=S0038-23612017000200008&lng=en>.
- Vismer HF, Morrison JGL, ‘Mycetoma caused by Actinomadura (Streptomyces) madurae’, S Afr Med J. 1974, 48, 433-437.

**WESTERN AFRICA**

**Burkina Faso**

- Diallo B, Andonaba JB, Konaté I, ‘Mycétome actinomycosique extrapodal plurifocal : bonne réponse au traitement par l'association cotrimoxazole et AINS’, J Mycol Med. 2015, 25(4), 297-302.
- Ouédraogo NA, Tiemtore-Kambou BMA, Ouédraogo NA et al, ‘Interest of Moringa oleifera in the Treatment of Fungal Mycetoma’, JOJ Case Stud. 2017, 2(5). Available at doi: 10.19080/JOJCS.2017.02.555600
- Sakande B, Traore SS, Kabore J et al, ‘Parasitic Diseases in Burkina Faso. An Histopathological Study’, Bull Soc Path Ex. 1998, 91(3), 217-20.
- Savadogo M, Boushab MB, Sondo KA et al, ‘Un pied de Madura résistant au traitement médical’, Med. Afr. Noire. 2014, 61(4), 220-222.

**Côte d’Ivoire**

- Adoubryn KD, Koffi KE, Troh E et al, ‘Les mycétomes autochtones de Côte d’Ivoire: caractères épidémiologiques et étiologiques des cas conﬁrmés’, J Mycol Med. 2009, 19, 71-76.

**Guinea**

- Cabrita J, ‘Human mycoses in Portugal [1960–1973]’, Mycopathologia. 1974, 54(3), 347-360.
- Desoubeaux G, Millon A, Freychet B et al, ‘Eumycetoma of the foot caused by Exophiala jeanselmei in a Guinean woman’, J Mycol Med. 2013, 23(3), 168-175.

**Guinea-Bissau**

- Salas-Coronas J, ‘Micetoma por Fusaarium solani’, Rev Clin Esp. 2011, 211(3), e16-17.

**Liberia**

- Camain R, Segretain G, Nazimoff O, ‘Maduromycosis in Senegal and Mauritania; epidemiological aspects and histopathological study’, Sem Hop. 1957, 33(13, 2), 771-774.

**Mali**

- Ahmed A, van de Sande W, Verbrugh H et al, ‘Madurella mycetomatis strains from mycetoma lesions in Sudanese patients are clonal’, J Clin Microbiol. 2003, 41(10), 4537–4541. Available at doi:10.1128/jcm.41.10.4537-4541.2003
- Gentilini M, ‘Primary inguino-perineal localisation of mycetoma with black grains in an African originating from Mali’, Bull Soc Path Ex. 1968, 61(1), 112-121.
- Hind B, Badredine H, ‘Actinomycetoma of the arm disseminated to the chest wall’, Pan Afr Med J. 2015, 20, 306. Available at doi:10.11604/pamj.2015.20.306.5766
- Machmachi H, Godnieau N, Develoux M et al, ‘Black grain mycetoma caused by Leptosphaeria tompkinsii’, Med Mycol. 2011, 49(2), 186-189.
- Mahe A, Develoux M, Lienhardt C et al, ‘Mycetomas in Mali: causative agents and geographic distribution’, Am J Trop Med Hyg. 1996, 54(1), 77-79.

**Mauritania**

- Andreu JM, ‘Actual treatment of fungus mycetoma: interest in associating ketoconazole and conservative surgery’, Med Trop (Mars). 1986, 46, 3.
- Ravisse P, Huerre M, De Bièvre C et al, ‘Les mycétomes en Mauritanie’, J Mycol Méd. 1992, 2, 154-159.

**Niger**

- Audoin J, Romanet JP, Rusterholtz B, ‘Surgical therapy in African mycetoma. Indications a propos of 160 cases’, Med Trop (Mars). 1986, 46(3), 283-292.
- Camain R, Segretain G, Nazimoff O, ‘Maduromycosis in Senegal and Mauritania; epidemiological aspects and histopathological study’, Sem Hop. 1957, 33(13, 2), 771-774.
- Develoux M, Audoin J, Treguer J et al, ‘Mycetoma in the Republic of Niger: clinical features and epidemiology’, Am J Trop Med Hyg. 1988, 38(2), 386-390.
- Develoux M, Vetter JM, Audoin J et al, ‘63 cases of mycetoma in the Niger Republic (etiological study based on histopathology)’, Bull Soc Pathol Exot Filiales. 1985, 78(5), 574-584.

**Nigeria**

- Agarwal SC, Mathur DR, ‘Mycetoma in northern Nigeria’, Trop Geogr Med. 1985, 37(2), 133-135.
- Gugnani HC, Suselan AV, Anikwe RM, ‘Actinomycetoma in Nigeria’, J Trop Med Hyg. 1981, 84, 259-263.
- Jacyk WK, Lawande RV, Tulpule SS. ‘Deep mycoses in West Africa: a report of 13 cases and review of the Nigerian literature.’ J Natl Med Assoc. 1981, 73(3), 251-6. Available at <https://www.ncbi.nlm.nih.gov/pmc/articles/PMC2609798/>
- Kalil M, Ekanem IOA, Gugnani HC et al, ‘Some deep mycoses diagnosed by histopathology in South Eastern Nigeria’, Rev Iberoam Micol. 1999, 16, 221-224.
- Onuigbo WIB, Cugnani HC, ‘Deep mycoses prevalent in the Igbos of Nigeria’, Int J Dermatol. 1976, 15, 432-437.
- Samaila MO, Abdullahi K, ‘Cutaneous manifestations of deep mycosis: An experience in a tropical pathology laboratory’, Indian J Dermatol [serial online]. 2011, 56, 282-286. Available at: <http://www.e-ijd.org/text.asp?2011/56/3/282/82481>
- Visvanathan R, ‘Surgical treatment of fungal mycetoma’, Can J Surg. 1989 32(1), 74-76.

**Senegal**

- Baylet R, Camain R, Chabal J et al, ‘Nouvelle contribution à l’étude des mycétomes au Sénégal’, Bull Soc Med Afr Noire Lang Fr. 1968, 13(2), 311-313.
- Beketi AK, Ba MC, Sy MH et al, ‘Le mycétome crânio-cervical’, Neurochirurgie. 2005, 51(5), 471-475. Available at: doi 10.1016/s0028-3770(05)83505-2
- Bèzes H, ‘L’aspect chirurgical des mycétomes à Dakar’, J Chir (Paris). 1961, 82, 13-32.
- Champeau MF, ‘A propos des mycétomes à grains noirs de l’A. O. F. (Signification du grain)’, Ann. Parasitol. Hum. Comp. 1954, 29(1, 2), 135-147. Available at: https://doi.org/10.1051/parasite/1954291135
- Diatta BA, Ndiaye M, Diop A et al, ‘Un mycétome fongique tumoral dorsal: intérêt de la chirurgie large associée à la terbinafine’, J Mycol Med. 2014, 24(4), 351-354. Available at: doi 10.1016/j.mycmed.2014.10.015
- Dieng MT, Niang SO, Diop B et al, ‘Actinomycétomes au Sénégal. Étude de 90 cas’, Bull Soc Path Ex. 2005, 98(1), 14-17.
- Dieng MT, Sy MH, Diop BM et al, ‘Mycetoma: 130 cases’, Vestn Dermatol Venerol. 2003, 130(1), 16-19.
- Diongue K, Boye A, Diallo MA et al, ‘Dermatophytic mycetoma of the scalp due to an atypical strain of Microsporum audouinii identified by MALDI-TOF MS and ITS sequencing’, J Mycol Med. 2019 (online), available at: doi 10.1016/j.mycmed.2019.03.001
- Diouf B, Fustee R, Pouye I et al, ‘Formes anatomo-cliniques des mycetomes a Dakar’, Bull Soc Med Afr Noire Lang Fr. 1965, 10(4), 564-588.
- Gueye NN, Seck SM, Diop Y et al, ‘Orbital mycetoma: a case report’, J Fr Ophtalmol. 2013, 36(5), 435-441.
- N’Diaye B, Dieng MT, Perez A et al, ‘Clinical efficacy and safety of oral terbinafine in fungal mycetoma’, Int J Dermatol. 2006, 45, 154-157.
- Ndiaye D, Ndiaye M, Sène PD et al, ‘Mycétomes diagnostiqués au Sénégal de 2008 à 2010’, J Mycol Med. 2011, 21(3), 173-181.
- Ndiaye M, Diatta BA, Diallo M et al, ‘Une présentation atypique tumorale d'un mycétome actinomycosique dorsolombaire’, J Mycol Med. 2014, 24(1), 44-47. Available at: https://doi.org/10.1016/j.mycmed.2013.10.002
- Sarr L, Niane MM, Diémé CB et al, ‘Chirurgie des mycétomes fongiques à grain noir. À propos de 44 patients pris en charge à l’Hôpital Aristide le Dantec de Dakar (Sénégal) de décembre 2008 à mars 2013’, Bull Soc Path Ex. 2016, 109(1), 8-12.
- Seye SI, Bassene N, Sy H et al, ‘Mycetoma of the fingers. Apropos of 5 case reports’, Ann Chir Main. 1988, 7(4), 322-326. Available at https://www.ncbi.nlm.nih.gov/pubmed/3233044
- Strobel M, Ndiaye B, Marchand JP et al, ‘Red grain mycetomas (A. pelletieri). Apropos of 20 new cases in Dakar’, Bull Soc Pathol Exot Filiales. 1981, 74(2), 155-63. Available at: doi: 10.1016/j.mycmed.2011.07.003

**Togo**

- Darré T, Saka B, Mouhari-Toure A et al, ‘Mycetoma in the Togolese: An Update from a Single-Center Experience’, Mycopathologia. 2018, 183(6), 961-965. Available at: https://doi.org/10.1007/s11046-018-0260-y
- Pitche P, Napo-Koura G, Kpodzro K et al, ‘Les mycétomes au Togo, Aspects épidémiologiques et étiologiques des cas histologiquement diagnostiqués’, Méd. Afr. Noire. 1999, 46(6), 322-325. Available at: <https://www.researchgate.net/publication/242774196_LES_MYCETOMES_AU_TOGO_Aspects_epidemiologiques_et_etiologiques_des_cas_histologiquement_diagnostiques>

**EASTERN ASIA**

**China**

- Chen B, Zhu L, Xuan X et al, ‘Isolation of both Pseudozyma aphidis and Nocardia otitidiscaviarum from a mycetoma on the leg’, Int J Dermatol. 2011, 50, 714-719. Available at doi:10.1111/j.1365-4632.2010.04814.x
- Chen X, Liu L, Zhang W, ‘Case of mycetoma caused by Nocardia asteroides’, Chin J Derm Venerol. 2009, 23(8), 509-510.
- Liang J, Yang H, Lui Z et al, ‘Mycetoma caused by Nocardia asteroides’, J Clin Dermatol. 2006, 35(10), 650-651.
- Wang R, Yao X, Li R, ‘Mycetoma in China: A Case Report and Review of the Literature’, Mycopathologia. 2019, 184(2), 327-334. Available at https://doi.org/10.1007/s11046-019-00324-z
- Wang S, Yang Q, Ye H, ‘Image Gallery: Actinomycetoma caused by Gordonia terrae in an immunocompetent woman’, Br J Dermatol. 2018, 179, e90. Available at doi:10.1111/bjd.16728
- Wang X, Zhou T, Deng D et al, ‘A Case of Cutaneous Nocardiosis with Involvement of the Trachea, Anterior Mediastinum and Sternum’, Case Rep Dermatol. 2010, 2, 177-182. Available at: https://www.karger.com/Article/Pdf/321635
- Xiujiao X, Hong S, Ai-e X, ‘Eumycetoma due to Acremonium falciforme acquired in China’, Mycoses. 2012, 55, e4-7. Available at doi:10.1111/j.1439-0507.2011.02059.x
- Yan J, Deng J, Zhou CJ et al, ‘Phenotypic and molecular characterization of Madurella pseudomycetomatis sp. nov., a novel opportunistic fungus possibly causing black-grain mycetoma’, J Clin Microbiol. 2010, 48(1), 251–257. Available at doi:10.1128/JCM.00018-09

**Japan**

- Fukushiro R, Mariat F, ‘Note on a mycetoma due to Nocardia caviae seen in Japan’, Bull Soc Pathol Exot Filiales. 1965, 58(2), 185-188.
- Gyotoku T, Kayashima K, Nishimoto K et al, ‘Cutaneous nocardiosis developing around gravel inserted during a traffic injury’, J Dermatol. 2002, 29(12), 803-9. Available at https://www.ncbi.nlm.nih.gov/pubmed/12532048
- Hoshino Y, Mukai A, Yazawa K et al, ‘Transvalencin A, a Thiazolidine Zinc Complex Antibiotic Produced by a Clinical Isolate of Nocardia transvalensis’, J Antibiot. 2004, 57(12), 797-802. Available at https://doi.org/10.7164/antibiotics.57.797
- Ichinomiya A, Nishimura K, Takenaka M et al, ‘Mycetoma caused by Nocardia transvalensis with repeated local recurrences for 25 years without dissemination to viscera’, J Dermatol. 2014, 41, 556-557. Available at doi:10.1111/1346-8138.12496
- Kashima M, Kano R, Mikami Y et al, ‘A successfully treated case of mycetoma due to Nocardia veterana’, Br J Dermatol. 2005, 152, 1349-1352. Available at doi:10.1111/j.1365-2133.2005.06551.x
- Mukai A, Fukai T, Matsumoto Y et al, ‘Transvalencin Z, a New Antimicrobial Compound with Salicylic Acid Residue from Nocardia transvalensis IFM 10065’, J Antibiot. 2006, 59, 366-369. Available at https://www.nature.com/articles/ja200653
- Ouchi K, Sato T, Yoshizawa N et al, ‘A case of cutaneous Pseudallescheria boydii infection after trauma’, Jpn J Med Mycol. 2008, 49(2), 119-123. Available at https://doi.org/10.3314/jjmm.49.119
- Sakayama K, Kidani T, Sugawara Y et al, ‘Mycetoma of Foot: A Rare Case Report and Review of the Literature’, Foot Ankle Int. 2004, 25(10), 763-767. Available at https://doi.org/10.1177/107110070402501012
- Sato T, ‘Practical management of deep cutaneous fungal infections’, Med Mycol J. 2017, 58E, e71-77. Available at https://www.jstage.jst.go.jp/article/mmj/58/2/58_17.006/_pdf/-char/ja
- Shimizu A, Ishikawa O, Nagai Y et al, ‘Primary cutaneous nocardiosis due to Nocardia nova in a healthy woman’, Br J Dermatol. 2001, 145, 154-156. Available at doi:10.1046/j.1365-2133.2001.04302.x
- Uenotsuchi T, Moroi Y, Urabe K et al, ‘Cutaneous Scedosporium apiospermum infection in an immunocompromised patient and a review of the literature’, Acta Derm Venereol. 2005, 85(2), 156-159. Available at doi: 10.1080/00015550410024553

**Republic of Korea**

- Seol CA, Sung H, Kim DH et al, ‘The First Korean Case of Disseminated Mycetoma Caused by Nocardia pseudobrasiliensis in a Patient on Long-Term Corticosteroid Therapy for the Treatment of Microscopic Polyangiitis’, Ann Lab Med. 2013, 33(3), 203-207. Available at <https://doi.org/10.3343/alm.2013.33.3.203>

**SOUTHERN ASIA**

**India**

- Bakshi R, Mathur DR, ‘Incidence and changing pattern of mycetoma in western Rajasthan’, Indian J Pathol Microbiol. 2008, 51, 154-155. Available at http://www.ijpmonline.org/text.asp?2008/51/1/154/40433
- Bhat RM, Monteiro RC, Bala N et al, ‘Subcutaneous mycoses in coastal Karnataka in south India’, Int J Dermatol. 2016, 55, 70-78. Available at doi:10.1111/ijd.12943
- Chufal SS, Thapliyal NC, Gupta MK, ‘An approach to histology-based diagnosis and treatment of Madura foot’, J Infect Dev Ctries. 2012, 6, 684-688. Available at https://doi.org/10.3855/jidc.2387
- Grueber HL, Kumar TM, ‘Mycetoma caused by Streptomyces somaliensis in north India’, Sabouraudia. 1970, 8(2), 108-111.
- Hazra B, Bandyopadhyay S, Saha SK et al, ‘A study of mycetoma in eastern India’, J Commun Dis. 1998, 30(1), 7-11. Available at https://www.ncbi.nlm.nih.gov/pubmed/9842158
- Maiti PK, Ray A, Bandyopadhyay S, ‘Epidemiological aspects of mycetoma from a retrospective study of 264 cases in West Bengal’, Trop. Med. Int. 2002, 7, 788-792. Available at doi:10.1046/j.1365-3156.2002.00915.x
- Masoodi Z, Mansoor T, Ali WM et al, ‘Actinomycetoma leg ulcers in north India’, Wounds. 2014, 26(5), 147-55.
- Sanghvi A, Sharma JC, Joshi KR et al, ‘Etiology & distribution of mycetoma in Rajasthan, India’, Indian J Med Res. 1987, 85, 694-698. Available at http://www.ijmr.in/CurrentTopicView.aspx?year=Indian+J+Med+Res+85%2c+June+1987%2c+pp+694-698%24Original+Article
- Sawatkar GU, Wankhade VH, Supekar BB et al, ‘Mycetoma: A Common Yet Unrecognized Health Burden in Central India’, Indian Dermatol Online J. 2019, 10(3), 256–261. Available at doi:10.4103/idoj.IDOJ_358_18
- Talwar P, Sehgal SC, ‘Mycetomas in North India’, Sabouraudia. 1979, 17(3), 287-291. Available at https://doi.org/10.1080/00362177985380421
- Venugopal PL, Venugopal TL, ‘Actinomadura madurae causing mycetomas in Madras’, Indian J Pathol Microbiol. 1991, 34(2), 119-125. Available at https://www.ncbi.nlm.nih.gov/pubmed/1752637
- Venugopal TV, Venugopal PV, Paramasivan CN et al, ‘Mycetomas in Madras’, Sabouraudia. 1977, 15(1), 17-22. Available from doi: 10.1080/00362177785190041

**Iran**

- Asgari M, Alilou M, ‘Mycetoma in Iran. The first report of eight cases with mycological studies’, Ann Soc Belg Med Trop. 1972, 52(3), 287-305.
- Bassiri-Jahromi S, ‘Mycetoma in Iran: Causative agents and geographic distribution’, Indian J Dermatol. 2014, 59, 529. Available at http://www.e-ijd.org/text.asp?2014/59/5/529/139889
- Mahmoudabadi AZ, Zarrin M, ‘Mycetomas in Iran: a review article’, Mycopathologia. 2008, 165, 135. Available at https://doi.org/10.1007/s11046-007-9066-z

**Pakistan**

- Mirza SH, Campbell C, ‘Mycetoma caused by Nocardia transvalensis’, J Clin Pathol. 1994, 47, 85-86. Available at <https://jcp.bmj.com/content/jclinpath/47/1/85.full.pdf>

**Sri Lanka**

- Cooray GH, ‘Fungal infections in Ceylon’, Ceylon Med J. 1962, 7, 185-96.
- Douwes KE, Schmalzbauer E, Hans-Jörg L et al, ‘Branched filaments no fungus, ovoid bodies no bacteria: Two unusual cases of mycetoma’, J Am Acad Dermatol. 2003, 49(2), S170-173. Available at doi: 10.1067/mjd.2003.302
- Gunathilake R, Perera P, Sirimanna G, ‘Curvularia lunata: a rare cause of black-grain eumycetoma’, J Mycol Med. 2014, 24(2), 158-160. Available at doi: 10.1016/j.mycmed.2013.09.005
- Ratnaike VT, Yoganathan M, Suntharalingam M et al, ‘Two cases of mycetoma’, Ceylon Med J. 1969, 14(1), 32-34.

**SOUTH-EASTERN ASIA**

**Cambodia**

- André M, Brumpt V, Destombes P et al, ‘Fungal mycetoma with black grains due to Pyrenochaeta romeroi in Cambodia’, Bull Soc Pathol Exot Filiales. 1968, 61(1), 108-112.
- Tournier Lasserve C, Sok Heang Sun, Despruniee J, ‘Osseous mycosis caused by mycetoma with black granules in Cambodia’, Med Trop (Mars). 1965, 25(5), 646-648.

**Indonesia**

- de Hoog GS, Buiting A, Tan CS et al, ‘Diagnostic problems with imported cases of mycetoma in The Netherlands’, Mycoses. 1993, 36, 81-87. Available at doi: 10.1111/j.1439-0507.1993.tb00693.x
- de Hoog GS, van Diepeningen AD, Mahgoub ES et al, ‘New Species of Madurella, Causative Agents of Black-Grain Mycetoma’, J Clin Microbiol. 2012, 50(3), 988-994. Available at doi: 10.1128/JCM.05477-11

**Lao People’s Democratic Republic**

- Rattanavong S, Vongthongchit S, Bounphamala K et al, ‘Actinomycetoma in SE Asia: the first case from Laos and a review of the literature’, BMC Infect Dis. 2012, 12, 349. Available at <https://doi.org/10.1186/1471-2334-12-349>
- Vongphoumy I, Dance DAB, Dittrich S et al, ‘Case Report: Actinomycetoma Caused by Nocardia aobensis from Lao PDR with Favourable Outcome after Short-Term Antibiotic Treatment’, PLoS Negl Trop Dis. 2015, 9(4), e0003729. Available at https://doi.org/10.1371/journal.pntd.0003729

**Malaysia**

- Burns-Cox CJ, ‘Maduromycosis in the forearm in a Bajau native of Sabah’, Med J Malaya. 1965, 19(4), 318-319.
- Geh GS, ‘A case of madura foot successfully treated with large doses of intravenous crystalline penicillin’, Med J Malaya. 1969, 24(2), 147-50.
- Ponnampalam JT, ‘The genus cephalosporium as a cause of Madura foot in Mayala’, Med J Malaya. 1964, 18, 229-231.

**Philippines**

- Abad-Venida LA, Reyes VO, ‘Eumycetoma (Phialemonium spp) cured by terbinafine’, J Phil Soc Cut Med. 2001, 2(1), 8-11.
- Bocobo F, De Leon D, Reyes A, ‘Black grain maduromycosis. (First case reported in the Philippines’, J Philipp Med Assoc. 1960, 36(5), 345-350.
- Cardones ARG, Frez LF, ‘Actinomycetoma secondary to Nocardia brasiliensis: a case report and review’, J Phil Dermatol Soc. 2001, 7(1), 12-18.
- Cerna EMAD, Montinola FL, ‘Eumycetoma in a Filipino adult man’, J Phil Dermatol Soc. 2009, 18(2), 41-43.
- Dumdum AI, Banate-Gulfan GO, Ledesma TGV et al, ‘Stage III eumycetoma successfully treated with oral ketoconazole and surgical debulking’, J Phil Dermatol Soc. 2016, 25(1), 58-61.
- Handog EB, Dayrit JF, ‘Mycology in the Philippines, revisited’, Jpn J Med Mycol. 2005, 46, 71-76. Available at http://www.jsmm.org/common/jjmm46-2_071.pdf
- Reyes AC, ‘A contribution to the study of mycetoma in the Philippines: maduromycosis caused by monosporium apiospermum (laboratory studies)’, Acta Med Philipp. 1963, 19, 89-102.
- Reyes AC, Tangco AG, Punsalang AP, ‘Maduromycosis (maduromycotic mycetoma) in the Philippines caused by Madurella grísea’, Southeast Asian J Trop Med Public Health. 1960, 57, 1216.
- Reyes AC, Tangco AG, Punsalang AP, ‘Maduromycosis (maduromycotic mycetoma) in the Philippines caused by Madurella grisea’, Southeast Asian J Trop Med Public Health. 1971, 2(1), 17-21.
- Sutantoyo CJ, Dayrit JF, Gabriel TG et al, ‘Mistaken identity: Eumycetoma Masquerading as Squamous Cell Carcinoma’, J Phil Dermatol Soc. 2018, 27(1), 81-3.

**Thailand**

- Kotrajaras R, ‘Mycetoma, a review of seventeen cases seen at the Institute of Dermatology, Bangkok, Thailand’, J Dermatol. 1981, 8(2), 133-137.
- Mahaisavariya P, Chaiprasert A, Apichati Sivayathorn D, ‘Deep fungal and higher bacterial skin infections in Thailand: clinical manifestations and treatment regimens’, Int J Dermatol. 1999, 38, 279-284. Available at doi:10.1046/j.1365-4362.1999.00681.x
- Supanakorn S, Rungruxsirivorn S, Patradul A et al, ‘Head and neck mycetoma’, J Med Assoc Thai. 1998, 81(12), 1019-1022. Available at http://www.jmatonline.com/index.php/jmat/article/viewfile/3647/3602
- Thianprasit M, Sivayathorn A, ‘Black Dot Mycetoma’, Mycoses. 1984, 27, 219-226. Available at doi:10.1111/j.1439-0507.1984.tb02022.x
- Youngchaiyud U, Thasnakorn P, Chantarakul N et al, ‘Maduromycosis of the hand due to Phialophora jeanselmei’, Southest Asian J Trop Med Pub Hlth. 1972, 3(1), 138-142. Available at <http://www.tm.mahidol.ac.th/seameo/1972-3-1/1972-3-1-138.pdf>

**Timor-Leste**

- Townell N, Locke T, Gibbons M et al, ‘Mycetoma in Timor-Leste and first report of nocardiosis’, Infect Dis Rep. 10(3). Available at <https://doi.org/10.4081/idr.2018.7804>

**Vietnam**

- Freland C, Fur JL, Nemirovsky-Trebucq B et al, ‘Primary cutaneous nocardiosis caused by Nocardia otitidiscaviarum: two cases and a review of the literature’, J Trop Med Hyg. 1995, 98, 395-403.

**WESTERN ASIA**

**Israel**

- Alteras I, Abraham D, Ideses C et al, ‘Mycetoma of the forearm due to Actinomadura madurae’, Mycopathologia. 1988, 103(55), 55-57. Available at https://doi.org/10.1007/BF00437222
- Alteras I, Feuerman EJ, Dayan I, ‘Mycetoma Due to Nocardia Caviae’, International Journal of Dermatology. 1980, 19, 260-262. Available at doi:10.1111/j.1365-4362.1980.tb00325.x
- Alteras I, Hagler J, Trattner A et al, ‘Mycetoma with black granules: first case in a native of Israel’, Hautarzt. 1992, 43(7), 446-7.
- Bitan O, Weiner-Well Y, Segal R et al, ‘Mycetoma (Madura Foot) in Israel: Recent Cases and a Systematic Review of the Literature’, Am J Trop Med Hyg. 2017, 96(6), 1355-1361.
- Dostrovsky A, Raubitschek F, Sagher F, ‘A case of black grain mycetoma caused by a Madurella species’, Dermatologica. 1952, 104, 415.
- Tamir G, Adler A, Hagler J et al, ‘Mycetoma of the foot - surgical treatment with free flap reconstruction’, Eur J Plast Surg. 1995, 18, 124-126.

**Lebanon**

- Brounst G, Nakib A, ‘Premier cas de mycétome ou pied de madura isole au liban dû à Streptomyces Nocardia asteroids’, Rev Med Moyen Orient. 1963, 20, 563-4.
- Saarinen KA, Lestringant GG, Czechowski J et al, ‘Cutaneous Nocardiosis of the Chest Wall and Pleura – 10-Year Consequences of a Hand Actinomycetoma’, Dermatology. 2001, 202, 131-133.

**Saudi Arabia**

- Al Gannass, ‘A Chronic Madura foot: mycetoma and/or Actinomyces spp or actinomycosis’, Case Reports. 2018. Available at https://casereports.bmj.com/content/2018/bcr-2018-224859
- Al-Ali AA, Kashgari TQ, Nathani PG et al, ‘Radiological Manifestations of Madura Foot in the Eastern Province of Saudi Arabia’, Ann Saudi Med. 1997, 17(3), 298-301.
- Al‐Tawfiq JA, ‘Chronic foot swelling with purulent discharge’, Int J Dermatol. 2012, 52, 1595-1596. Available at https://doi.org/10.1111/j.1365-4632.2011.05151.x
- Al‐Tawfiq JA, Amr SS, ‘Madura leg due to Exophiala jeanselmei successfully treated with surgery and itraconazole therapy’, Medical Mycology. 2009, 47(6), 648-652. Available at <https://doi.org/10.1080/13693780802669194>
- Anim JT, el-Gaali NO, ‘Mycetoma of the scalp: report of a case’, Trans R Soc Trop Med Hyg. 1986, 80(3), 412-4. Available at doi: 10.1016/0035-9203(86)90328-7
- Bendl BJ, Mackey D, Al-Saati F et al, ‘Mycetoma in Saudi Arabia’, J Trop Med Hyg. 1987, 90, 51-59.
- Mufti ST, Aljhdali H, ‘Mycetoma at a tertiary care hospital in Saudi Arabia: correlation of histopathological and clinical findings’, Asian Pac J Trop Biomed. 2015, 5(4), 331-336.
- Sibany A, Al-Mashat F, Meccawi AA et al, ‘Mycetoma in the Western Region of Saudi Arabia’, J KAU Med Sci. 1999, 7(2), 87-94.
- Venugopal PV, Venugopal TV, Laing WN et al, ‘Etiology and Epidemiology of Mycetomas in Saudi Arabia, Ann Saudi Med. 1990, 10(6), 611-614. Available at <https://doi.org/10.5144/0256-4947.1990.611>

**Turkey**

- Elmaci I, Senday D, Silav G et al, ‘Nocardial Cerebral Abscess Associated with Mycetoma, Pneumonia, and Membranoproliferative Glomerulonephritis’, J Clin Microbiol. 2007, 45(6), 2072-2074. Available at doi: 10.1128/JCM.01358-06
- Erbakan N, Or AN, Unal M et al, ‘A review of mycetomas in Turkey’, Mycopathol Mycol Appl. 1973, 51(1), 105-13.
- Gündüz K, Örgüç S, Demireli P et al, ‘A case of mycetoma successfully treated with itraconazole and co‐trimoxazole’, Mycoses. 2006, 49, 436-438. Available at doi:10.1111/j.1439-0507.2006.01260.x
- Hoog GS, Buiting A, Tan CS et al, ‘Diagnostic problems with imported cases of mycetoma in The Netherlands’, Mycoses. 1993, 36, 81-87. Available at doi:10.1111/j.1439-0507.1993.tb00693.x
- Kalender AM, Baykan H, Özkan F et al, ‘Negative Pressure Wound Therapy and Skin Graft in Madura Foot Treatment’, Balkan Med J. 2012, 99, 214-217. Available at doi: 10.5152/balkanmedj.2012.014

**Yemen**

- Clemow FG, ‘Mycetoma (Madura foot) in the Yemen’, Br Med J. 1906, 2364, 918-919. Available at doi: 10.1136/bmj.1.2364.918
- Khatri ML, Al‐Halali HM, Fouad Khalid M et al, ‘Mycetoma in Yemen: clinicoepidemiologic and histopathologic study’, Int J Dermatol. 2002, 41, 586-593. Available at doi:10.1046/j.1365-4362.2002.01383.x
- Landau Z, Feld S, Frumkin A et al, ‘Nocardia brasiliensis skin infections’, Isr J Med Sci. 1986, 22(5), 397-399. Available at <https://www.ncbi.nlm.nih.gov/pubmed/3744789>
- Oyston JK, ‘Madura foot: a study of twenty cases’, J Bone Joint Surg. 1961, 43B(2), 259-267. Available at <https://online.boneandjoint.org.uk/doi/pdf/10.1302/0301-620X.43B2.259>
- Yu AM, Zhao S, Nie LY, ‘Mycetomas in Northern Yemen: Identification of Causative Organisms and Epidemiologic Considerations’, Am J Trop Med Hyg. 1993, 48(6), 812-817. Available at https://doi.org/10.4269/ajtmh.1993.48.812

**EASTERN EUROPE**

**Bulgaria**

- Balabanoff VA, ‘Les mycétomes du sud-est de la Bulgarie’, Ann Parasitol Hum Comp. 1980, 55(5), 605-613. Available at <https://www.parasite-journal.org/articles/parasite/pdf/1980/05/parasite1980555p605.pdf>

**Hungary**

- Tóth EJ, Nagy GR, Homa M et al, ‘Recurrent Scedosporium apiospermum mycetoma successfully treated by surgical excision and terbinafine treatment: a case report and review of the literature’, Ann Clin Microbiol Antimicrob. 2017, 16(31), 1-9. Available at doi: 10.1186/s12941-017-0195-z

**Romania**

- Avram A, ‘Sur les agents causaux des mycétomes d‘une contrée à climat tempéré. A propos de 23 cas roumains’, Mykosen. 1969, 12(1), 39-47.

**SOUTHERN EUROPE**

**Albania**

- Buonfrate D, Gobbi F, Angheben A et al, ‘Autochthonous Cases of Mycetoma in Europe: Report of Two Cases and Review of Literature’, PLoS One. 2014, 9(6), e100590. Available at <https://doi.org/10.1371/journal.pone.0100590>
- De Palma L, Marinelli M, Pavan M, ‘A rare European case of Madura Foot due to actinomycetes’, Joint Bone Spine. 2006, 73(3), 321-324. Available at doi: 10.1016/j.jbspin.2005.10.018
- Fida M, Saraceno R, Gjylametaj N et al, ‘Eumycetoma Pedis in an Albanian Farmer’, Cutis. 2018, 102(5), E13-E15. Available at https://www.ncbi.nlm.nih.gov/pubmed/30566558
- Rigopoulos D, Mavridou M, Nicolaidou E et al, ‘Mycetoma due to actinomycetes: a rare entity in Europe’, Int J Dertmatol. 2000, 39, 557-558. Available at doi:10.1046/j.1365-4362.2000.00819-5.x

**Croatia**

- Marinovic I, Branica H, ‘The first known case of mycetoma (fungus) pedis – Madura foot in Dalmatia’, Lijec Vjesn. 1986, 108(9), 376-379.

**Greece**

- Douwes KE, Schmalzbauer E, Hans-Jörg L et al, ‘Branched filaments no fungus, ovoid bodies no bacteria: Two unusual cases of mycetoma’, J Am Acad Dermatol. 2003, 49(2), S170-173. Available at doi: 10.1067/mjd.2003.302
- Ispoglou SS, Zormpala A, Androulaki A et al, ‘Madura foot due to Actinomadura madurae: Imaging appearance’, J Clin Imaging Sci. 2003, 27, 233-235. Available at doi: 10.1016/S0899-7071(02)00502-8
- Pelzer K, Tietz HJ, Sterry W et al, ‘Isolation of both Sporothrix schenckii and Nocardia asteroides from a mycetoma of the forefoot’, Br J Dermatol. 2001, 143, 1311-1315. Available at doi:10.1046/j.1365-2133.2000.03908.x

**Italy**

- Ajello L, Padhye AA, Chandler FW et al, ‘Fusarium moniliforme, a new mycetoma agent restudy of a European case‘, Eur J Epidemiol. 1985, 1(1) ,5-10. Available at <https://doi.org/10.1007/BF00162306>
- Binazzi M, Papini M, ‘Madura Foot by Actinomadura madurae/ A Further Report on an Autochthonous Italian Case’, Mycoses. 1983, 26, 298-304. Available at doi:10.1111/j.1439-0507.1983.tb03214.x
- Mencarini J, Antonelli A, Scoccianti G et al, ‘Madura foot in Europe: diagnosis of an autochthonous case by molecular approach and review of the literature’,  New Microbiol. 2016, 39(2), 156-159. Available at <http://www.newmicrobiologica.org/PUB/allegati_pdf/2016/2/156.pdf>
- Nazzaro G, Veraldi S, ‘Mycetomas: The Experience of the Dermatology Unit of the University of Milan’, Dermatopathology. 2018, 5, 6-9. Available at doi: 10.1159/000486236
- Posteraro P, Frances C, Didona B, ‘Persistent subcutaneous Scedosporium apiospermum infection’, Eur J Dermatol. 2003, 13, 603-605.
- Satta R, Sanna S, Cottoni F, ‘Madurella infection in an immunocompromised host’, Int J Dermatol. 2008, 39, 939-941. Available at doi:10.1046/j.1365-4362.2000.00988-6.x
- Usai F, Carluccio A, Caddeo R, ‘Madura foot: descrizione di un caso clinico’, Microbiologia Medica. 2005, 20(3), 161. Available at <https://doi.org/10.4081/mm.2005.3501>

**WESTERN EUROPE**

**Portugal**

- Brandao FN, Figuiredo MM, ‘Micetoma do pé pela “Nocardia asteroides”’, J Medico. 1963, 52, 401-404.
- Cabrita J, Figueiredo MM, ‘Movimento micológico excluindo dermatófitos (1965–1970)’, Trab Soc Port Derm Vener. 1971, 29, 91–101.
- Castellani A, De Brito MM, Pinto MR, ‘An actinomycete isolated from an autochthonous case of mycetoma in Portugal’, J Trop Med Hyg. 1959, 62(2), 27-36.
- Nobre G, ‘Micetoma com lesões metásticas por Nocardia asteroides’, Trab Soc Port Derm Vener. 1970, 28, 123–128.

**France**

- Degavre B, Joujoux JM, Dandurand M et al, ‘First report of mycetoma caused by Arthrographis kalrae: Successful treatment with itraconazole’, J Am Acad Dermatol. 1997, 37(2), 318-320. Available at 10.1016/s0190-9622(97)70016-1
- Gilquin JM, Riviere B, Jurado V et al, ‘First Case of Actinomycetoma in France Due to a Novel Nocardia Species, Nocardia boironii sp. nov.’, mSphere. 1(6), e0030916. Available at doi:10.1128/mSphere.00309-16
- Yera H, Bougnoux ME, Jeanrot C, et al ‘Mycetoma of the foot caused by Fusarium solani: identification of the etiologic agent by DNA sequencing’, J Clin Microbiol. 2003, 41(4), 1805–1808. Available at doi:10.1128/jcm.41.4.1805-1808.2003

**Germany**

- Horré R, Schumacher G, Marklein G et al, ‘Mycetoma due to Pseudallescheria boydii and co-isolation of Nocardia abscessus in a patient injured in road accident’, Med Mycol. 2002, 40(5), 525-527. Available at <https://doi.org/10.1080/mmy.40.5.525.527>

**LATIN AMERICA AND THE CARIBBEAN**

**Antigua and Barbuda**

- Zoutman DE, Sigler L. ‘Mycetoma of the foot caused by Cylindrocarpon destructans’, J Clin Microbiol. 1991, 29(9), 1855–1859. Available at <https://www.ncbi.nlm.nih.gov/pmc/articles/PMC270224/>

**Cuba**

- Ferrer RN, Perez JY, Garcia OVR, ‘Micetoma. Presentación de un caso’, Rev Cubana Ortop Traumatol. 2001, 15(1, 2). Available at http://scielo.sld.cu/scielo.php?script=sci_arttext&pid=S0864-215X2001000100015
- Pardo-Castello V, Trespalacios F, ‘Superficial and Deep mycoses in Cuba’, South Med J. 1959, 52(1), 7-15.
- Resnik BI, Burdick AE, ‘Improvement of eumycetoma with itraconazole’, J Am Acad Dermatol. 1995, 33(5), 917-919. Available at <https://doi.org/10.1016/0190-9622(95)90437-9>

**Curaçao**

- de Vries GA, van der Hoeven LH, ‘Report of a case of black grain maduromycosis of the foot caused by an aberrant strain of madurella mycetomi (Laveran) brumpt on curacao, Netherlands Antilles’, Mycopathologia. 1957, 8(4), 253-259. Available at <https://doi.org/10.1007/BF02052295>

**Dominican Republic**

- Chadfield HW, ‘Maduromycosis’, Proc R Soc Med. 1964, 57(2), 103-105. Available at <https://www.ncbi.nlm.nih.gov/pmc/articles/PMC1897775/>
- Salcedo-Irizarry F, Vélez-Rosario R, Marcos-Martínez MJ et al, ‘Madura foot (mycetoma), an unusual fungal infection in Puerto Rico’, poster, University of Puerto Rico- School of Medicine.
- Santiago Sánchez-Mateos JL, Moreno Izquierdo R, Jiménez Navarro BC, ‘Subcutaneous tumor in foot after trauma with acacia thorn’, Rev Clin Esp. 2007, 207(10), 527-9.

**Guadeloupe**

- Segretain G, Destombes P, Proye G et al, ‘Premier cas de mycétome fongique à grains blancs en Guadeloupe. Probabilité d’un nouvel agent de maduromycose’, Bull Soc Path Ex. 1967, 1, 13-20. Available at <http://www.pathexo.fr/documents/articles-bull/BullSocPatholExot-1967-60-1-013-020.pdf>

**Haiti**

- Mollica PW, Brenner MA, Nair SR et al, ‘Madura Foot: a case report’, Cutis. 1981, 27(6), 634-635, 643-644, 659.
- Rouphael NG, Talati NJ, Franco-Paredes C, ‘A painful thorn in the foot: a case of eumycetoma’, Am J Med Sci. 2007, 334(2), 142-144.

**Jamaica**

- Fletcher CL, Moore MK, Hay RJ, ‘Eumycetoma due to Madurella mycetomatis acquired in Jamaica’, Br J Dermatol. 2001, 145(6), 1018-1021. Available at https://www.ncbi.nlm.nih.gov/pubmed/11899126
- Hay RJ, MacKenzie DWR, ‘Mycetoma (madura foot) in the United Kingdom–a survey of forty‐four cases’, Clin Exp Dermatol. 1983, 8, 553-562. Available at doi:10.1111/j.1365-2230.1983.tb01823.x
- Pilsczek FH, Augenbraun M, ‘Mycetoma fungal infection: multiple organisms as colonizers or pathogens?’, Rev Soc Bras Med Trop. 2007, 40(4), 463-465. Available at http://www.scielo.br/pdf/rsbmt/v40n4/a17v40n4.pdf

**Martinique**

- Vezon G, Desbois N, Boisseau-Garsaud AM et al, ‘Microsporum canis mycetoma of the scalp’, Ann Dermatol Venereol. 2000, 127(8-9), 729-31. Available at <https://www.ncbi.nlm.nih.gov/pubmed/11011164>

**Montserrat**

- Hay RJ, ‘Mycoses imported from the West Indies. A report of three cases’, Postgrad Med J. 1979, 55, 603-604. Available at <https://pmj.bmj.com/content/postgradmedj/55/647/603.full.pdf>

**Puerto Rico**

- Carrión AL, ‘Estudio micologico de un caso de Micetoma por Cephalosporium en Puerto Rico’, Mycopathologia. 1939, 2(2), 165-170. Available at <https://doi.org/10.1007/BF00444048>

**Saint Kitts and Nevis**

- Anning, La Touche, Hunter, ‘Madura foot (mycetoma)’, Br J Dermatol. 1958, 70, 309-310. Available at doi: 10.1111/j.1365-2133.1958.tb13347.x

**Trinidad and Tobago**

- Brownell I, Pomeranz M, Ma L, ‘Eumycetoma’, Dermatol Online J. 2005, 11(4), 10. Available at <https://escholarship.org/uc/item/80p2w3ds>

**United States Virgin Islands**

- Carrión AL, Knott J, ‘Micetoma Producido por Monosporium Apiospermum en la Pequeña Antilla de Santa Cruz’, Puerto Rico J Pub Health. 1944, 20(1), 100-107.

**CENTRAL AMERICA**

**Guatemala**

- Mayorga R, Close de Leon JE, ‘Sur Une Souche de Madurella grisea Sporifere Isolee D'un Mycetome Guatemalteque a Grains Noirs’, Sabouraudia. 1966, 4(4), 210-214.
- Silva AS, Torre LP, Pena JD et al, ‘Lesión cutánea de larga evolución en paciente inmunocompetente’, Enferm Infecc Microbiol Clin. 2013, 31(8), 555-556.

**Mexico**

- Bonifaz A, Tirado-Sanchez A, Calderon L et al, ‘Mycetoma: experience of 482 cases in a single center in Mexico’, PLoS Negl Trop Dis. 2014, 8(8), e3102. Available at doi:  10.1371/journal.pntd.0003102
- Buot G,  Lavalle P,  Mariat F et al, ‘Epidemiologic study of mycetomas in Mexico. Apropos of 502 cases’, Bull Soc Pathol Exot Filiales. 1987, 80(3), 329-339. Available at https://europepmc.org/abstract/med/3621395
- Cruz COF, Vega SDC, Ramírez HL et al, ‘Mycetoma. A Report of 174 Cases Studied in 30 Years in a General Hospital in Mexico City (1987-2017)’, Derma Cosmética y Quirúrgica. 2018, 6(4), 263-267. Available at <https://www.medigraphic.com/cgi-bin/new/resumenI.cgi?IDARTICULO=84508>
- Guerra‐Leal JD, Medrano‐Danés LA, Montemayor‐Martinez A et al, ‘The importance of diagnostic imaging of mycetoma in the foot’, Int J Dermatol. 2019, 58, 600-604. Available at doi:10.1111/ijd.14351
- López Martínez R, Méndez Tovar LJ, Lavalle P et al, ‘Epidemiology of mycetoma in Mexico: study of 2105 cases’, Gac Med Mex. 1992, 128(4), 477-481.
- López-Martínez R, Méndez-Tovar LJ, Bonifaz A et al, ‘Update on the epidemiology of mycetoma in Mexico. A review of 3933 cases’, Gac Med Mex. 2013, 149(5), 586-592.
- Sánchez-Herrera K, Sandoval H, Couble A et al, ‘Phenotypic and genotypic evaluation of 18 Nocardia isolates from human clinical samples in Mexico’, J Mycol Med. 2012, 22(1), 1-7. Available at doi: 10.1016/j.mycmed.2011.10.008

**Panama**

- Calero MC, ‘Madura foot (mycetoma) first report from the Isthmus of Panama’, Arch Derm Syphilol. 1947, 55(6), 761-71.

**SOUTH AMERICA**

**Argentina**

- Biagini RE, Martínez TE, Museli A et al, ‘Mycetoma in northern Argentina’, Med Cutan Ibero Lat Am. 1983, 11(6), 431-436.
- Luque AG, Mujica MT, D’Anna ML et al, ‘Micetoma podal por fusarium solani’, Boletín Micológico. 1991, 6(1-2), 55-57. Available at https://doi.org/10.22370/bolmicol.1991.6.1-2.1609
- Negroni R, ‘Contribución al estudio de los micetomas en la República Argentina’, Med Cut ILA. 1974, 2(5), 353-362.
- Negroni R, Daneri GL, Arechavala A et al, ‘Estudio clínico y microbiológico de los micetomas observados en el Hospital de Infecciosas Francisco J. Muñiz en el período 1989-2004’, Rev argent microbiol. 2006, 38(1). Available at <http://www.scielo.org.ar/scielo.php?script=sci_arttext&pid=S0325-75412006000100004>
- Niño FL, ‘Actinomycosic mycetoma in Argentina’, Sem Med. 1950, 27(29), 117-119.
- Niño FL, Freire RS, ‘Maduromycotic mycetoma in the province of Chaco (Argentina)’, Mycopathol Mycol Appl. 1966, 28(1), 95-6.
- van Gelderen de Komaid A, ‘Physiological and pathogenic characteristics of Nocardia brasiliensis isolated from human mycetomas’, Mycopathologia. 1989, 105(2), 111-116. Available at https://doi.org/10.1007/BF00444033
- Zapater RC, ‘Maduromycosis in Argentina’, Rev Asoc Med Argent. 1958, 72(7), 254-258.

**Bolivia**

- Negroni R, Daneri GL, Arechavala A et al, ‘Estudio clínico y microbiológico de los micetomas observados en el Hospital de Infecciosas Francisco J. Muñiz en el período 1989-2004’, Rev argent microbiol. 2006, 38(1). Available at <http://www.scielo.org.ar/scielo.php?script=sci_arttext&pid=S0325-75412006000100004>

**Brazil**

- Braga FJHN, Araujo EB, Camargo EE et al, ‘Scintigraphic evaluation of mycetoma’, Nucl Med Commun. 1993, 14, 814-818.
- Castro LG, Belda Júnior W, Salebian A et al, ‘Mycetoma: a retrospective study of 41 cases seen in São Paulo, Brazil, from 1978 to 1989’, Mycoses. 1993, 36(3-4), 89-95. Available at https://www.ncbi.nlm.nih.gov/pubmed/8366881
- Castro LG, Piquero‐Casals J, ‘Clinical and mycologic findings and therapeutic outcome of 27 mycetoma patients from São Paulo, Brazil’, Int J Dermatol. 2008, 47, 160-163. Available at doi:10.1111/j.1365-4632.2008.03447.x
- Lacaz CDS, ‘Distribuição geográfica dos micetomas no Brasil’, An Bras Dermatol. 1978, 56(3). Available at <http://www.anaisdedermatologia.org.br/detalhe-artigo/2005/Distribuicao-geografica-dos-micetomas-no-Brasil>
- Sampaio FMS, Wanke B, Freitas DFS et al, ‘Review of 21 cases of mycetoma from 1991 to 2014 in Rio de Janeiro, Brazil’, PLoS Negl Trop Dis. 2017, 1-18. Available at doi: 10.1371/journal.pntd.0005301

**Chile**

- Filippi J, Quezada F, Lagos M, ‘Micetoma por Actinomadura madurae en el pie. Reporte de un caso en Chile’, Rev Méd Chile. 2008, 136, 1448-1452. Available at <https://scielo.conicyt.cl/pdf/rmc/v136n11/art11.pdf>
- Jerez R, Schafer F, Fich F et al, ‘Actinomycotic mycetoma due to Actinomadura madurae’, Rev Chilena Infectol. 2012, 29(4), 459-463. Available at <https://scielo.conicyt.cl/scielo.php?script=sci_arttext&pid=S0716-10182012000400018&lng=en&nrm=iso&tlng=en>

**Colombia**

- Cárdenas V, Calle G, Cortés C et al, ‘Micetomas: Presentacion de dos nuevos casos y revision del tema’, Antioquia Med. 1966, 16(2), 117-132.
- Pena CE, ‘Deep mycotic infections in Colombia’, Am J Clin Pathol. 1967, 47(4), 505-520.

**Paraguay**

- Negroni R, Daneri GL, Arechavala A et al, ‘Estudio clínico y microbiológico de los micetomas observados en el Hospital de Infecciosas Francisco J. Muñiz en el período 1989-2004’, Rev argent microbiol. 2006, 38(1). Available at <http://www.scielo.org.ar/scielo.php?script=sci_arttext&pid=S0325-75412006000100004>

**Peru**

- Bustamante B, Ahmed SA, De Hoog GS et al, ‘Phaeoacremonium sphinctrophorum as a Novel Agent of Eumycetoma’ JAMA Dermatol. 2016, 152(9), 1063–1065. Available at doi:10.1001/jamadermatol.2016.1310
- Lim W, Eadie K, Horst-Kreft D, ‘VNTR confirms the heterogeneity of Madurella mycetomatis and is a promising typing tool for this mycetoma causing agent’, Med Mycol. 2019, 57(4), 434-440. Available at <https://doi.org/10.1093/mmy/myy055>

**Uruguay**

- Conti-Diaz IA, ‘Mycetoma and premycetomatous lesions in Uruguay’, Mycopathologia. 1980, 72(1), 59-64.

**Venezuela**

- Borelli D, ‘Pyrenochaeta mackinnonii, a new species causing mycetoma’, Castellania. 1976, 4, 227-234.
- De Albornoz MB, ‘Cephalosporium serrae, agente etiologico de micetomas’, Mycopathologia. 1974, 54, 485-498.
- De Albornoz MB, ‘MICETOMA (Pie de Madura) DEBIDO A CEPHALOSPORIUM RECIFEI’, Derm Venez. 1964, 4, 56-64.
- Maaz TB, ‘MICETOMA PRODUCIDO POR EL MONOSPORIUM APIOSPERMUM’, Derm Venez. 1967, 6(3, 4), 155-158.
- Padin C, Fernández-Zeppenfeldt G, Yegres F et al, ‘Scytalidium dimidiatum: hongo oportunista para el hombre y árboles de Mangifera indica en Venezuela’, Rev Iberoam Micol. 2005, 22(3), 172-173. Available at doi: 10.1016/s1130-1406(05)70035-8
- Pérez-Blanco M, Hernández-Valles R, Fernández-Zeppenfeldt G et al, ‘Mycetoma: report of 3 cases in Falcón State, Venezuela’, Invest Clin. 1996, 37(1), 61-73.
- Rojas OC, León‐Cachón RB, Moreno‐Treviño M et al, ‘Molecular identification of unusual Mycetoma agents isolated from patients in Venezuela’, Mycoses. 2017, 60, 129-135. Available at doi:10.1111/myc.12579

**NOTHERN AMERICA**

**Canada**

- Adami JG, Kirkpatrick RC, ‘A case of Madura foot disease’, Tr Assn Am Phys. 1895, 43, 2-9. Available at https://archive.org/details/cihm_11539/page/n11

**United States of America**

- - Bartlett MS, Tight RR. 'Actinomycetoma in the United States', Rev. Infect. Dis., 1981; 3(6), 1139-1150. Available at<https://doi.org/10.1093/clinids/3.6.1139>
  - Berd D. ‘Nocardia brasiliensis Infection in the United States', Am J Clin Pathol, 1972, 59, 254-258.
  - Bergeron JR, Mullins JF, Ajello L, ‘Mycetoma Caused by Nocardia pelletieri in the United States’, Arch Derm. 1969, 99, 564-566.
  - Boyd MF, Crutchfield ED, ‘A contribution to the study of mycetoma in North America’, Am J Trop Med. 1921, 1, 215.
  - Carroll DS, ‘Mycetoma pedis’, Radiology. 1949, 53, 81-84.
  - Foltz KD, Fallat LM. 'Madura foot: atypical finding and case presentation', J Foot Ankle Surg. 2004, 43(5), 327-31.
  - Gammel JA, Miskdjan H, Thatcher HS, ‘Madura foot (mycetoma)’, Arch Derm & Syph. 1926, 13, 66.
  - Gay DM, Bigelow JB, ‘Madura Foot Due to Monosporium Apiospermum in a Native American’, Am J Pathol. 1930, 6(3), 325-336.
  - Green R, Bolton TC, Woolsey AB. 'Mycetoma - Madura Foot', Ann Surg, 1948, 128(5), 1015-1022.
  - Green WO. ‘Mycetoma in the United States’, Am J Clin Pathol. 1964, 42(1), 75-91.
  - Guy WH, ‘Nocardiosis cutis resembling sporotrichosis’, Arch Dermatol Syph. 1920, 2(2), 137-143.
  - Halde C, Padhye AA, Haley LD et al, ‘Acremonium Falciforme as a cause of mycetoma in California’, Sabouraudia. 1976, 14, 319-326.
  - Halde C, Ringrose EJ, ‘Mycetoma originating in northern California’, Arch Dermatol. 1956, 74, 80-85.
  - Halloran CR, ‘Mycetoma in an American negro’, Arch Dermatol Syph. 1927, 16, 611-612.
  - Hanlon TJ, Gephardt MC, Hopps HC, ‘Mycetoma in the United States: review with report of a case from new area’, J Okla State Med Assoc. 1955, 48(10), 299-303.
  - Hopps S, Roach A, Yuen C, et al. 'Treatment for a eumycetoma infection caused by Aspergillus in an immunocompromised host: a case report', Transpl Infect Dis. 2014, 17(1), 94-97. Available at <https://doi.org/10.1111/tid.12321>
  - Hyde JN, Senn N, ‘A contribution to the study of mycetoma of the foot as it occurs in America’, J Genit Syst Disord. 1896, 14(1), 1-15.
  - Jones JW, Alden HS, ‘Maduromycotic mycetoma (Madura foot)’, JAMA. 1931, 96(4), 256-260.
  - Kemper GWH, ‘A case of podelcoma’, Am Fam Physician. 1876, 14, 129-135.
  - Kish LS, Taylor JS, Bergfield WF et al, ‘Petriellidium (Allescheria) boydii mycetoma in an immunosuppressed host’, Cleve Clin J Med. 1983, 50(2), 209-211.
  - Lamb JH, Kelly F, Shackelford PO et al, ‘Pregnenolone acetate in treatment of mycetoma (nocardiosis)’, Arch Dermatol Syph. 1950, 62, 58-65.
  - Long PI, Campana HA, ‘An unusual mycetoma’, Arch Dermatol. 1966, 93, 341-345.
  - Martinez RE, Couchel S, Swartz WM et al. 'Mycetoma of the hand', J Hand Surg. 1989, 14(5), 909-912. Available at<https://doi.org/10.1016/S0363-5023(89)80102-9>
  - Mohr JA, Muchmore HG, ‘Maduromycosis due to Aleescheria boydii’, JAMA. 1968, 204(4), 125-126.
  - Moore M, Lane CW, Gaul LE, ‘Nocardiosis of the knee caused by Nocadia brasiliensis’, Arch Dermatol Syph. 1954, 70, 302-310.
  - Neuhauser E, ‘Black grain maduromycosis caused by Madurella grisea’, AMA Arch Derm. 1955, 72(6), 550-555.
  - Pagenstecher GA, ‘Madura foot, more properly called mycetoma’, JAMA. 1922, 78(18), 1363-1365.
  - Peters JT, ‘A clinical cure of Madura foot’, Am J Trop Med Hyg. 1945, S1-25 (4), 363 – 365.
  - Puestow KL, ‘Maduromycosis’, Arch Derm & Syph. 1929, 20, 642.
  - Roe LD, Stone OJ, Graham JH, ‘Nocardia brasiliensis mycetoma’, Arch Dermatol. 1975, 111, 1371-1372.
  - Seabury JH, McGinn M, Salvaggio J, ‘Mycetoma mandibularis due to Nocardia pelletieri’, Am J Med, 1973, 55(6), 846-850. DOI: 10.1016/0002-9343(73)90267-2
  - Shaw RM, MacGregor JW, ‘Maduromycosis: with the report of a case due to monosporium apiospermum’, CMAJ. 1935, 33(1), 23–28.
  - Stierstorfer MB, Schwartz BK, Mcguire JB et al.  ‘Pseudallescheria boydii Mycetoma in Northern New England.’ Int. J. Dermatol. 1988, 27, 383-387. DOI:[10.1111/j.1365-4362.1988.tb02382.x](https://doi.org/10.1111/j.1365-4362.1988.tb02382.x)
  - Sutton RL, ‘Mycetoma in America’, JAMA. 1913, 60(18), 1339-1342.
  - Symmers D, Sporer A, ‘Maduromycosis of the hand’, Arch Path. 1944, 37, 309.
  - Twining HE, Dixon HM, Weidman FD, ‘Penicillin in treatment of madura foot’, Bull U S Army Med Dep. 1946, 46(3), 417-429.
  - Van Etta LL, Peterson LR, Gerding DN, ‘Acremonium falciforme (Cephalosporium falciforme) mycetoma in a renal transplant patient’, Arch Dermatol. 1983, 119(8), 707-8.
  - Warintarawej A, Winter WG Jr, Goodman NL, ‘Maduromycosis (Madura foot) in Kentucky.’, South Med J, 1975, 68(12), 1570-1575. Available at<https://www.ncbi.nlm.nih.gov/pubmed/1239084>
  - Winslow R, ‘Fungous diseases of the foot, or Madura foot, in America’, Ann Surg. 1917, 66(4), 496-498.

**OCEANIA**

**Australia**

- - Abbott L, Hunter I, Cutler G et al, ‘Mycetoma of the foot caused by Nocardia brasiliensis’, Med J Aust. 1972, 1, 1137-1139.
  - Dementjeva GR, ‘Studies on a case of actinomycetoma pedis in Queensland’, Sabourdaudia. 1970, 8, 81-92.
  - Leech PJ, Bedbrook GM, ‘An Australian case of maduromycosis’, Aust N Z J Surg. 1970, 39, 293-295.
  - Lucas RE, Armstrong PK, ‘Two cases of mycetoma due to Nocardia brasiliensis in central Australia’, Med J Aust. 2000, 172, 167-169.

**New Caledonia**

- - Huerre M, ‘Les mycoses profondes observes en Nouvelle-Calédonie’, Bull Soc Path Ex. 1991, 84, 247-256.
  - Monchy D, Huerre MR, de Bièvre C, ‘Deep‐seated fungal diseases in the South Pacific, especially in New Caledonia’, Mycoses. 1998, 41, 31-34.

**Papua New Guinea**

- - Lytton DG, ‘Mycetoma in PNG with special reference to a case of extradural mycetoma’, Papua New G Med J. 1975, 18(1), 61-65.
  - Mitjà O, Hays R, Van Straten C et al, ‘Mycetoma Caused by Nocardia yamanashiensis, Papua New Guinea’, Am J Trop Med Hyg. 2012, 86(6), 1043-1045.

**Vanuatu**

- - McCormack JG, McIntyre PB, Tilse MH et al, ‘Mycetoma associated with Acremonium falciforme infection’, Med J Aust. 1987, 147(4), 187-188.

**French Polynesia**

- - Segonne J, ‘Premier cas de mycétome fungique à Tahiti’, Bull Soc Path Ex. 1971, 64(2), 197-201.

**Wallis and Futuna Islands**

- - Monchy D, Huerre MR, de Bièvre C, ‘Deep‐seated fungal diseases in the South Pacific, especially in New Caledonia’, Mycoses. 1998, 41, 31-34.
